# Attenuated Nuclear Tension Regulates Progerin‐Induced Mechanosensitive Nuclear Wrinkling and Chromatin Remodeling

**DOI:** 10.1002/advs.202502375

**Published:** 2025-05-08

**Authors:** Ji‐Eun Park, Juhyeon Jo, Kun Xu, Sun‐Ah Lee, Seong‐Beom Han, YigJi Lee, Won‐Ki Cho, Bo Li, Soo Hyun Kim, Dong‐Hwee Kim

**Affiliations:** ^1^ KU‐KIST Graduate School of Converging Science and Technology Korea University Seoul 02841 South Korea; ^2^ Department of Engineering Mechanics Tsinghua University Beijing 100084 China; ^3^ Department of Biological Sciences Korea Advanced Institute of Science and Technology (KAIST) Daejeon 34141 South Korea; ^4^ Biomaterials Research Center Biomedical Research Division Korea Institute of Science and Technology (KIST) Seoul 02792 South Korea; ^5^ Department of Integrative Energy Engineering College of Engineering Korea University Seoul 02841 South Korea

**Keywords:** actomyosin contractility, chromatin remodeling, heterochromatin, Hutchinson–Gilford progeria syndrome, LINC complex, mechanosensation, nuclear deformation, nuclear tension, nuclear wrinkling, progerin, SUN1

## Abstract

Hutchinson–Gilford progeria syndrome, caused by a mutation in the *LMNA* gene, leads to increased levels of truncated prelamin A, progerin, in the nuclear membrane. The accumulation of progerin results in defective nuclear morphology and is associated with altered expression of linker of the nucleoskeleton and cytoskeleton complex proteins, which are critical for nuclear signal transduction via molecular coupling between the extranuclear cytoskeleton and lamin‐associated nuclear envelope. However, the molecular mechanisms underlying progerin accumulation‐induced nuclear deformation and its effects on intranuclear chromosomal organization remain unclear. Here, the spatiotemporal evolution of nuclear wrinkles is analyzed in response to variations in substrate stiffness using a doxycycline‐inducible progerin expression system. It is found that cytoskeletal tension regulates the onset of progerin‐induced nuclear envelope wrinkling and that the molecular interaction between SUN1 and LMNA controls the actomyosin‐dependent attenuation of nuclear tension. Genome‐wide analysis of chromatin accessibility and gene expression further suggests that an imbalance in force between the intra‐ and extranuclear spaces induces nuclear deformation, which specifically regulates progeria‐associated gene expression via modification of mechanosensitive signaling pathways. The findings highlight the crucial role of nuclear lamin–cytoskeletal connectivity in bridging nuclear mechanotransduction and the biological aging process.

## Graphical abstract

The premature aging‐related progerin leads to defective nuclear morphology and is associated with disrupted molecular coupling between the extranuclear cytoskeleton and lamin‐associated nuclear envelope. It is discovered that progerin expression reduces nuclear tension, forms nuclear wrinkling, and enhances chromatin dynamics, thereby regulating progerin‐induced mechanosensitive signaling pathways.

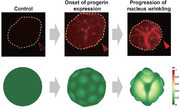

## 1 Introduction

Hutchinson–Gilford progeria syndrome (HGPS), a premature aging disease, is caused by a de novo point mutation (G608G; GGC > GGT) in the *LMNA* gene encoding lamins A and C, creating a truncated prelamin A form lacking 50 amino acid residues near the C terminus, commonly referred to as Δ50 lamin A or progerin.^[^ ^1^ ^]^ These amino acids include a cleavage site for the zinc metallopeptidase STE24 (ZMPSTE24), which is crucial in the formation of mature lamin A by removing the farnesyl group; loss of this cleavage site leads to a persistent farnesylation of progerin.^[^ ^2^ ^]^ Accumulation of progerin in the nuclear envelope (NE) compromises the structural and functional integrity of the nucleus, resulting in an abnormal nuclear shape, a hallmark of HGPS.^[^ ^3^ ^]^ For instance, fibroblasts obtained from patients with HGPS display defective nuclear shapes, including invaginations, lobulations, and wrinkles.^[^ ^3^, ^4^ ^]^ Furthermore, overexpression of Δ50 lamin A in primary dermal fibroblasts induces abnormalities in nuclear morphology similar to those observed in HGPS cells.^[^ ^5^ ^]^

Progerin expression also leads to altered molecular connections between the nucleus and cytoskeleton,^[^ ^6^ ^]^ further resulting in enhanced sensitivity to mechanical stress^[^ ^7^ ^]^ and reduced force propagation from the extracellular matrix (ECM) into the cell.^[^ ^8^ ^]^ ECM stiffness, which reflects tissue‐specific biomechanical properties, is detected by transmembrane receptor proteins, such as integrins,^[^ ^9^ ^]^ which mediates the formation of focal adhesion complexes. These complexes transmit mechanical signals into intracellular biochemical pathways, thereby inducing diverse cellular responses.^[^ ^10^ ^]^ Moreover, these adhesion complexes promote the polymerization of intracellular actin cytoskeletal networks, where actomyosin contractility‐induced mechanical forces allow cells to sense and respond to the biomechanical characteristics of their surrounding environment.^[^ ^11^ ^]^ These force‐transmitting molecular connections are largely mediated by the linker of the nucleoskeleton and cytoskeleton (LINC) complexes.^[^ ^12^ ^]^ The LINC complex is composed of a SUN (Sad1, UNC‐84) domain located in the inner nuclear membrane and a KASH (Klarsicht, ANC‐1, and Syne Homology) domain located in the outer nuclear membrane.^[^ ^13^ ^]^ Nuclear envelope spectrin‐repeat proteins (nesprins), consisting of actin‐binding N‐terminal and KASH domain‐binding C‐terminal domains, also bind to SUN proteins in the perinuclear space between the nuclear membranes.^[^ ^14^ ^]^ Thus, the LINC complex transmits biophysical stimuli into the nucleus through nesprin‐mediated molecular connections between the actin cytoskeleton and SUN proteins.^[^ ^15^ ^]^ In particular, LINC‐mediated cytoskeletal tension plays a key role in cellular mechanoresponses,^[^ ^16^ ^]^ where actomyosin contractility and force‐dependent reorganization of the cytoskeletal architecture remodel LINC‐associated protein components to transmit extracellular physical stimuli into the nucleus.^[^ ^17^ ^]^

Lamins interact with heterochromatic genomic regions via lamina‐associated domains (LADs) that are enriched with repressive histone marks, such as trimethylated histone H3 at lysine 9 (H3K9me2/3) and H3K27me3.^[^ ^18^ ^]^ Transcriptional repression of genes within such heterochromatic regions is attributed to the loss of DNA–nuclear lamina interactions that contribute to chromatin condensation and the expression of H3K9me2/3.^[^ ^19^ ^]^ Thus, lamin‐associated intranuclear chromatin organization correlates with nuclear stiffening, which is crucial for maintaining nuclear structural integrity in response to mechanical stress.^[^ ^20^ ^]^ Because NE‐associated SUN proteins directly bind chromatin to the nuclear periphery,^[^ ^21^ ^]^ impaired LINC complexes hinder intracellular force transmission, which, in turn, disrupts perinuclear cytoskeleton‐dependent mechanosensing of the extracellular microenvironment. Ultimately, mechanosensitive nuclear deformation compromises intranuclear elasticity distribution,^[^ ^22^ ^]^ indicating the remodeling of heterochromatin accessibility. Reduced chromatin mobility by the inhibition of actomyosin contractility or nuclear detachment from the cytoskeleton further highlights the critical role of an intact actomyosin apparatus and the LINC complex in mechanical signal transduction to chromatin.^[^ ^22^, ^23^ ^]^

Progerin expression disrupts the LINC complex by altering the expression of SUN proteins without affecting the localization of SUN1 and SUN2 to the nuclear membrane,^[^ ^24^ ^]^ resulting in altered cellular mechanotransduction.^[^ ^25^ ^]^ In NIH 3T3 fibroblasts expressing myc‐tagged progerin and in HGPS fibroblasts, enhanced SUN1 expression in response to progerin accumulation alters nuclear coupling to actin filaments (F‐actin) and microtubules (MT) through nesprin 2, resulting in defective nuclear movement and cell polarity.^[^ ^26^ ^]^ SUN1 overexpression in HGPS fibroblasts can further lead to increased SUN1–nesprin 2 coupling with MT, thereby inhibiting nuclear movement due to its complementary coupling with the actin cytoskeleton.^[^ ^26b^ ^]^ Consequently, nesprin 2‐mediated actomyosin tension applied to the NE is reduced,^[^ ^27^ ^]^ confirming a decrease in nuclear forces in response to SUN1 overexpression in progerin‐expressing cells. Moreover, SUN1 silencing in HGPS fibroblasts rescues the deformed nuclear morphology,^[^ ^28^ ^]^ whereas SUN2 depletion fails to restore progerin‐induced nuclear deformation.^[^ ^4^ ^]^ These observations highlight the role of SUN1 upregulation in nuclear deformation as a molecular hallmark of the pathological signature of HGPS.

Progerin expression increases F‐actin polymerization^[^ ^29^ ^]^ and RhoA activation,^[^ ^30^ ^]^ resulting in cytoskeletal stiffening.^[^ ^7^ ^]^ For instance, F‐actin polymerization is increased in mesenchymal stromal/stem cells from the *ZMPSTE24* ^−/−^ HGPS mouse model, Z24^−/−^ mesenchymal stromal cells (MSCs), which exhibit a higher elastic modulus than wild‐type MSCs. Increased RhoA activity in Z24^−/−^ MSCs is suppressed by pharmaceutical inhibition of RhoA signaling, resulting in reduced nuclear deformation.^[^ ^30^ ^]^ Consistent with the results from Z24^−/−^ MSCs, increased F‐actin polymerization and nuclear deformation in HGPS fibroblasts are reversed by treatment with a farnesyltransferase inhibitor.^[^ ^30^, ^31^ ^]^ Elevated cytoskeletal tension in progeria cells arises from mechanical stress due to both ECM stiffness^[^ ^32^ ^]^ and increased nuclear rigidity, as sustained RhoA signaling promotes F‐actin cytoskeletal stiffness.^[^ ^30^ ^]^ Progerin‐induced alterations in nucleocytoskeletal connections result in reduced propagation of cytoskeletal forces to the nucleus,^[^ ^8^ ^]^ as determined by particle tracking analysis in exogenous progerin‐expressing HeLa cells, human umbilical vein endothelial cells, and HGPS fibroblasts.^[^ ^8^ ^]^

Accumulating evidence suggests that nucleus‐responsive forces can alter epigenetic modifications through chromatin remodeling;^[^ ^20^ ^]^ mechanosensitive changes in forces applied to the nucleus can alter intranuclear heterochromatin reorganization and chromatin accessibility. For instance, dermal fibroblasts derived from patients with HGPS show changes in DNA methylation, and chromatin accessibility is enriched in NE‐associated regions, contributing to the abnormal gene expression patterns observed in HGPS.^[^ ^33^ ^]^ Therefore, progerin accumulation leads to widespread alterations in the repressive histone mark H3K27me3, disrupting the association between heterochromatin and the nuclear lamina in HGPS fibroblasts, resulting in the loss of chromosomal compartmentalization.^[^ ^34^ ^]^

Previously, studies on HGPS models have consistently demonstrated increased cytoskeletal tension and RhoA activation,^[^ ^30^ ^]^ which are essential to regulate force transmission to the nucleus. In turn, nuclear forces alter epigenetic modifications through chromatin remodeling.^[^ ^20^, ^33^ ^]^ However, the causal relationship between progerin‐induced nuclear–cytoskeletal force transmission and nuclear deformation in progerin‐expressing cells, as well as the consequent alterations in gene expression remains to be identified. While previous studies have shown that nuclear deformation can be regulated by overexpressed SUN1, the precise mechanism by which SUN1 overexpression induces nuclear deformation remains poorly understood. Therefore, here, we investigated the biophysical mechanisms that regulate nuclear morphological changes in response to progerin expression and their downstream effects on gene expression. To this end, we developed a human HGPS cell model, where the progerin expression was precisely controlled using the doxycycline‐induced Tet‐On system, overcoming the constraints of using the primary cells from patients with HGPS owing to their limited availability. We elucidated the mechanism by which progerin expression alters nuclear morphology over time. We further investigated how progerin accumulation at the nuclear membrane in HGPS cells affects LINC complex expression and the physical connectivity between the actin cytoskeleton and nuclear lamina. Furthermore, we examined the impact of these nuclear morphological changes on chromatin organization and the aberrant gene expression profiles in HGPS. Our study improves our understanding of the progerin‐induced alteration of nuclear tension that drives nuclear deformation.

## 2 Results

### 2.1 Mechanosensing of Substrate Stiffness Modulates Progerin‐Induced Nuclear Deformation

HGPS, characterized by abnormal nuclear morphology due to the expression of truncated prelamin A (Δ50 LMNA/progerin),^[^ ^35^ ^]^ displays impaired nuclear mechanotransduction.^[^ ^26b^ ^]^ To elucidate the molecular mechanism by which progerin accumulation alters mechanical signal transduction through nuclear deformation, we developed an HGPS model in HeLa cells, where the onset of progerin expression was precisely controlled by transfection with the XLone plasmid that combined the piggyBac transposon and Tet‐On 3G doxycycline‐inducible gene expression system (see the Experimental Section and Figure S1, Supporting Information).^[^ ^36^ ^]^

**Figure 1:**
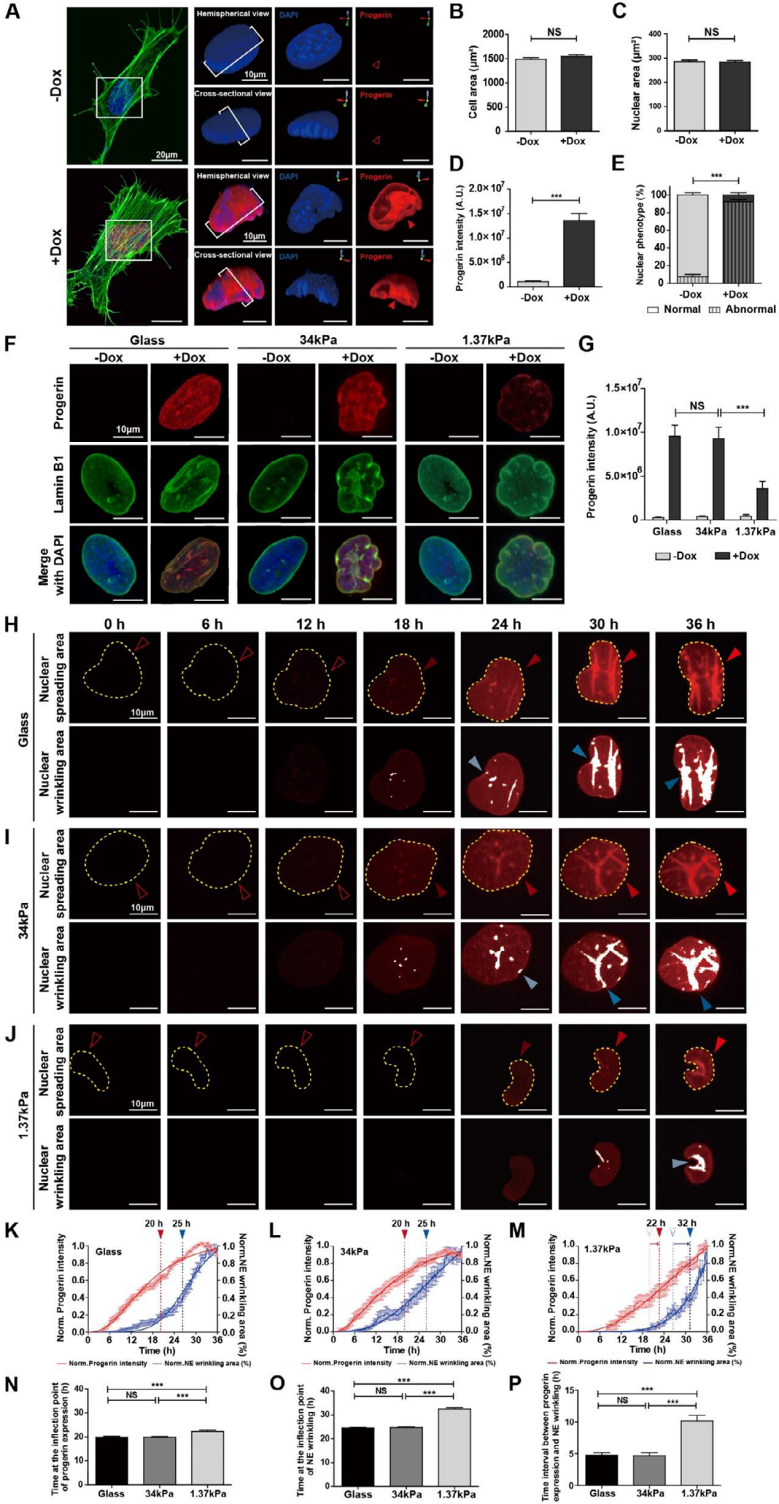
Substrate stiffness‐dependent differential evolution of nuclear deformation in doxycycline‐inducible progerin‐expressing HeLa cells. A–E) Morphological alterations of doxycycline‐controlled Tet‐On HeLa cells expressing mutant lamin A protein (Δ50 LMNA/progerin). Representative confocal images depict immunofluorescence staining for progerin (red), F‐actin (green), and nuclei (DAPI, blue) in doxycycline‐untreated control cells (−Dox) or doxycycline‐treated (2 µg mL^−1^) progerin‐expressing Tet‐On HeLa cells (+Dox). Hemispherical and cross‐sectional views of 3D‐rendered nuclei show progerin expression‐induced formation of abnormal nuclear morphology. Empty and full arrowheads indicate the absence and presence of progerin expression, respectively (A). Immunofluorescence intensity‐based quantifications of cell area (B), nuclear area (C), progerin expression (D), and the fractional occurrence of abnormal nuclear shapes (E) were performed in the absence and presence of doxycycline treatment. In panel B, 810 and 505 nuclei; in panel C, 138 and 153 nuclei; in panel D, 93 and 72 nuclei were analyzed under −Dox and +Dox conditions, respectively. For panel E, 70 to 105 nuclei were analyzed, which was independently repeated three times per each condition. Error bars indicate the standard error of the mean (S.E.M.); an unpaired *t*‐test was applied (***: *p* < 0.001, NS: not significant). F–G) Substrate stiffness‐dependent differential expression of progerin. Progerin (red), lamin B1 (green), and nuclei (DAPI, blue) of progerin‐expressing Tet‐On HeLa cells were plated on control glass substrates or polyacrylamide hydrogel (PAG) substrates with elastic moduli of 34 kPa (stiff) and 1.37 kPa (soft) (F). Control glass and stiff PAG substrates maintained doxycycline‐inducible progerin expression, while soft PAG substrates significantly reduced progerin expression (G). In panel G, >50 cells were tested per condition. Error bars indicate the S.E.M.; Student's *t*‐test was applied (***: *p* < 0.001, NS: not significant). H–J) Time‐lapse monitoring of substrate stiffness‐dependent progerin expression and nuclear deformation. Progerin intensity and nuclear envelope (NE) wrinkling were monitored every 20 min for 36 h in mCherry‐progerin‐expressing Tet‐On HeLa cells plated on control glass (H), stiff PAG (I), and soft PAG (J) substrates. Yellow dotted lines mark the nuclear boundary determined by differential interference contrast (DIC) imaging, showing nuclear spreading area. Full and empty arrowheads indicate the presence and absence of progerin (red) or NE wrinkling (blue), respectively; transparency of the full arrowheads represents the magnitude of progerin expression and NE wrinkling; oversaturated fluorescence intensity (white) indicates nuclear surface wrinkles (H–J). K–P) Quantifying differential onset of progerin expression and nuclear deformation in response to changes in substrate stiffness. Progerin expression and NE wrinkling were quantified by measuring fluorescence intensity (red curves) and the fraction of the nuclear spreading area occupied by NE wrinkling area (blue curves) after doxycycline treatment (K–M). All values were normalized using the formula (*x* – *x*_min_)/(*x*_max_ – *x*_min_) to range from 0 (min) to 1 (max). Progerin expression and NE wrinkling followed extended sigmoidal curves, with inflection points for progerin expression at 20 h and NE wrinkling at 25 h on control glass and stiff PAG substrates (K,L,N,O), which were delayed to 22 and 32 h, respectively, on soft PAG substrates (M–O). The time interval between inflection points of progerin expression and NE wrinkling extended from 5 h on control glass and stiff PAG substrates to 10 h on soft PAG substrates (P). In panels K–P, >20 cells were analyzed per condition. Error bars indicate the S.E.M.; one‐way analysis of variance (ANOVA) with Tukey's test was used for comparisons (***: *p* < 0.001, NS: not significant).

The treatment of Tet‐On gene‐expressing HeLa cells with 2 µg mL^−1^ doxycycline specifically induced progerin expression and dramatically increased the population of abnormal nuclei with blebs, lobulation, and wavy surface texture, while the overall cell and nuclear size remained unchanged (**Figure** **1**A–E; Figure S2A, Supporting Information). We noted that traditional nuclear morphometry measurements, including nuclear area, circularity, and aspect ratio (defined as nuclear spreading area, 4π(area)/(perimeter^2^), and the ratio of the longest axis to the perpendicular shortest axis, respectively), were insufficient to quantitatively assess the nuclear deformation induced by progerin expression (Figure 1B,C; Figure S2B,C, Supporting Information). Hence, we assessed the NE wrinkling area by measuring the fraction of the nuclear surface area occupied by NE wrinkles to more precisely represent progerin‐induced NE‐defective nuclei (Figure S2D, Supporting Information). To further confirm that doxycycline‐induced progerin expression controls the progression of nuclear deformation, we systematically quantified progerin expression and NE wrinkling at different time points, where we noted that the time‐dependent elevation of progerin expression preceded the increase in NE wrinkling (Figure S3, Supporting Information). These results demonstrate that our doxycycline‐controlled progerin‐expressing Tet‐On HeLa cell line not only replicates the deformed nuclear morphology detected in HGPS cells but also enables time‐dependent monitoring of progerin expression and nuclear deformation.

Mechanosensing of matrix rigidity alters protein expression by remodeling the nucleus–cytoskeletal connection, mediating the intracellular mechanical balance between cytoskeletal force and nuclear surface tension.^[^ ^37^ ^]^ Accordingly, we investigated whether cells adapted to differential substrate stiffness could modulate progerin expression in doxycycline‐activated HeLa cells (Figure 1F,G). Cells placed on control glass and polyacrylamide hydrogel (PAG) substrates, mimicking the in vivo elastic moduli of rigid and compliant organs,^[^ ^38^ ^]^ displayed deformed nuclear shapes in response to doxycycline‐induced progerin expression, regardless of substrate stiffness (Figure 1F). In contrast to doxycycline‐untreated cells not expressing progerin, doxycycline‐treated cells placed on rigid PAG substrates (*E* ≈ 34 kPa) maintained elevated progerin expression similar to cells placed on control glass substrates, while those grown on soft PAG substrates (*E* ≈ 1.37 kPa) showed a significant reduction in progerin expression (Figure 1G). This result strongly suggests that substrate stiffness‐dependent changes in intracellular tension could regulate progerin expression.

Since doxycycline‐controlled progerin expression led to nuclear deformation in a time‐dependent manner (Figure S3, Supporting Information), and progerin expression levels were altered by substrate stiffness (Figure 1F,G), we assessed whether differential substrate stiffness could modulate the temporal relationship between doxycycline‐induced progerin expression and nuclear deformation. To this end, we conducted real‐time live cell monitoring of mCherry‐tagged progerin‐expressing Tet‐On HeLa cells placed on substrates with varying stiffness (Figure 1H–J; Movies S1 and S2, Supporting Information). Time‐lapse imaging revealed that doxycycline‐induced progerin expression exhibited sigmoidal changes, followed by NE wrinkling, which was most evident on control glass substrates, but attenuated on reduced substrate stiffness (Figure 1H–M). Assessment of the inflection time points in the curves further revealed that progerin expression and nuclear deformation were delayed in cells placed on soft PAG substrates compared to those placed on control glass or stiff PAG substrates (Figure 1K–M). Specifically, the inflection points for progerin expression and NE wrinkling occurred at 20 and 25 h on control glass and stiff PAG substrates, respectively, whereas they were observed at 22 and 32 h on soft PAG substrates (Figure 1K–M). Consequently, the time interval between the inflection points of the curves for progerin expression and NE wrinkling was extended from 5 h on control glass and stiff PAG substrates to 10 h on soft PAG substrates (Figure 1N–P).

These results indicate that doxycycline‐induced progerin expression, followed by NE wrinkling, is highly mechanosensitive to changes in substrate stiffness, which further suggests that progerin‐induced nuclear deformation can be regulated by intracellular cytoskeletal tension applied to the nucleus.

### 2.2 Mechanosensing of Substrate Stiffness Induces the Differential Attenuation of Nuclear Tension in Progerin‐Expressing Cells

Previously, we demonstrated that substrate stiffness‐dependent spatial reorganization of lamin A/C is regulated by cytoskeletal tension,^[^ ^16^ ^]^ and that 3D morphological alterations of the nucleus in response to mechanical stimuli are regulated by nucleus–cytoskeletal connections.^[^ ^39^ ^]^ Because the LINC‐mediated molecular connection between the cytoskeleton and nuclear lamina facilitates force transmission across the nuclear membrane,^[^ ^21^ ^]^ we hypothesized that the nuclear tension between these elements could determine the substrate stiffness‐modulated differential onset of progerin‐induced nuclear deformation.

To directly measure the force applied to the nuclear–cytoskeletal connection, we used a fluorescence energy transfer (FRET)‐based NE tension sensor module tagging the inner nuclear membrane SUN‐binding domain and cytoplasmic F‐actin‐binding domain, mimicking the LINC complex associating nesprin 2^[^ ^27^ ^]^ (Figure S4A, Supporting Information). As an elevated FRET ratio in HGPS cells implies diminished nuclear tension due to reduced actomyosin contractility,^[^ ^27^ ^]^ we tested whether doxycycline‐induced progerin expression could impinge on the substrate stiffness‐dependent differential decline of nuclear tension. To monitor time‐dependent gradual changes in NE tension, we transiently transfected the nesprin tension sensor into progerin‐expressing Tet‐On HeLa cells before placing them on substrates of varying stiffness (Figure S4B,C, Supporting Information). We confirmed that the transfected nesprin tension sensors were specifically localized along the nuclear membrane and that their fluorescence intensity was maintained without significant decay during time‐lapse imaging of the nuclei every 6 h for 36 h (Figure S4D,E, Supporting Information).

**Figure 2:**
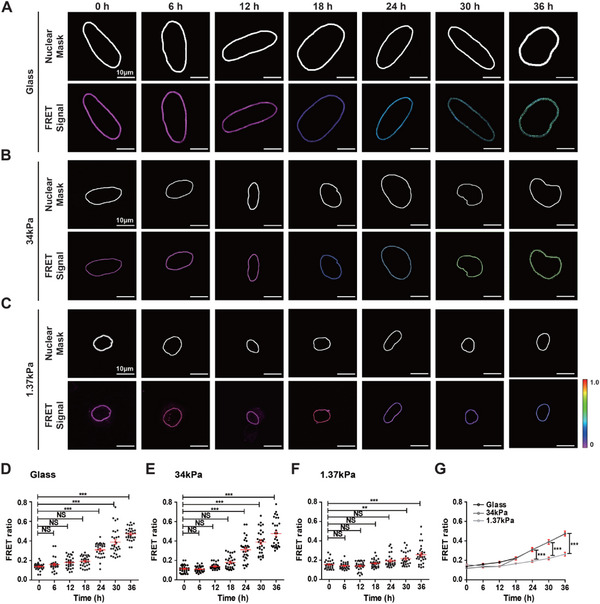
Substrate stiffness‐dependent differential NE tension. A–C) Visualization of substrate stiffness‐dependent changes in NE tension using the nesprin tension sensor. Tet‐On HeLa cells expressing fluorescence‐marker‐untagged progerin were transiently transfected with the nesprin tension sensor and plated on control glass or stiff (*E* ≈ 34 kPa) or soft (*E* ≈ 1.37 kPa) PAG substrates. NE tension was analyzed every 6 h for 36 h after doxycycline treatment. Binary layers outlining the nesprin tension sensor‐localized nuclear membrane were determined by creating polygonal hollow masks to exclude fluorescence intensity outside the nucleus (top rows). FRET signals were differentially color‐coded (bottom rows). Purple and red indicates high FRET efficiency. FRET efficiency, representing the inverse of NE tension, gradually increased in response to doxycycline‐induced progerin expression, but this rate was reduced in cells on soft PAG substrates compared to those on control glass and stiff PAG substrates (A and B vs C). D–G) Quantification of substrate stiffness‐modulated differential NE tension. Column scatter plots represent the time‐dependent increase in the FRET ratio in response to doxycycline‐induced progerin expression on control glass and stiff or soft PAG substrates. The nesprin tension sensor‐based FRET ratio increased significantly after 24 h in cells on glass (D) and stiff PAG substrates (E), but after 30 h in cells on soft PAG substrates (F). Accordingly, the FRET signal in cells on glass and stiff PAG substrates was significantly higher than in those on soft PAG substrates after 24 h (G). In panels D–G, red bars represent the mean ± S.D., and one‐way ANOVA with Tukey's test was applied for group comparisons (***: *p* < 0.001, **: *p* < 0.05, NS: not significant).

To analyze the substrate stiffness‐dependent differential application of NE tension in response to doxycycline‐induced progerin expression, we masked FRET signals located along the NE (**Figure** **2**A–C), thereby preventing interference with FRET signals stemming from other regions of the cell (see the Experimental Section for details). FRET efficiency was calculated by dividing the fluorescence intensity of the acceptor by that of the donor after background subtraction within the masked NE region, which was differentially color‐coded, where approaching purple indicated a decreased FRET ratio due to enhanced NE tension, and conversely, approaching red indicated an increased FRET ratio due to reduced NE tension (Figure 2A–C).

The FRET ratio, representing the opposite of NE tension, significantly increased in response to progerin expression, regardless of substrate stiffness, after 24 h of doxycycline treatment on control glass and stiff PAG substrates (Figure 2D,E), while the change was detected after 30 h on soft PAG substrates (Figure 2F). Interestingly, the temporal reduction in NE tension showed no difference between cells placed on glass and stiff PAG substrates (Figure 2D,E,G). Accordingly, a significant difference between the FRET ratios of cells placed on rigid and soft substrates was observed after 24 h (Figure 2G), which is consistent with a previous report showing that HGPS cells displayed lower nuclear tension than normal control cells.^[^ ^27^ ^]^

Together with our results quantifying the substrate stiffness‐dependent differential onset of progerin expression and nuclear deformation (Figure 1K–P), these data further reveal the temporal relationship between the progerin‐induced diminution of NE tension and nuclear deformation. We found that the time point at which nuclear tension was significantly reduced (i.e., a significantly increased FRET ratio) appeared between two distinct time points at which an abrupt increase in progerin expression and NE wrinkling were detected. This finding strongly supports the hypothesis that progerin induces nuclear deformation by reducing nuclear tension. In addition, we noted that the time interval between the increment of progerin expression and the reduction of nuclear tension, as well as the time interval between the reduction of nuclear tension and the increase in NE wrinkling, were doubled by relocating cells from the control glass and stiff PAG substrates to the soft PAG substrates, i.e., from 4 to 8 h and from 1 to 2 h, respectively.

These data indicated that the time required to decrease nuclear tension in response to progerin expression was approximately four times longer than that required to induce nuclear deformation due to reduced nuclear tension. Further, these findings strongly suggest that cytoskeletal tension, modulated by changes in substrate stiffness, mediates nuclear tension‐dependent nuclear deformation in progerin‐expressing cells.

### 2.3 Progerin‐Induced Reduction in Nuclear Tension Determines Mechanosensitive Nuclear Wrinkling

The temporal onset of progerin expression, reduction in NE tension, and nuclear wrinkling in response to doxycycline treatment were delayed by relocating cells from stiff to soft substrates (Figures 1 and 2), which suggest that not only gene expression driven by biochemical stimuli but also its biophysical consequences, such as alterations in NE tension and nuclear surface remodeling, are sensitive to the mechanical balance between the nucleus and the physical environment of the cell. Specifically, we employed a computational model to determine whether substrate stiffness‐dependent nuclear force mediates the differential evolution of progerin‐induced nuclear wrinkling.

**Figure 3:**
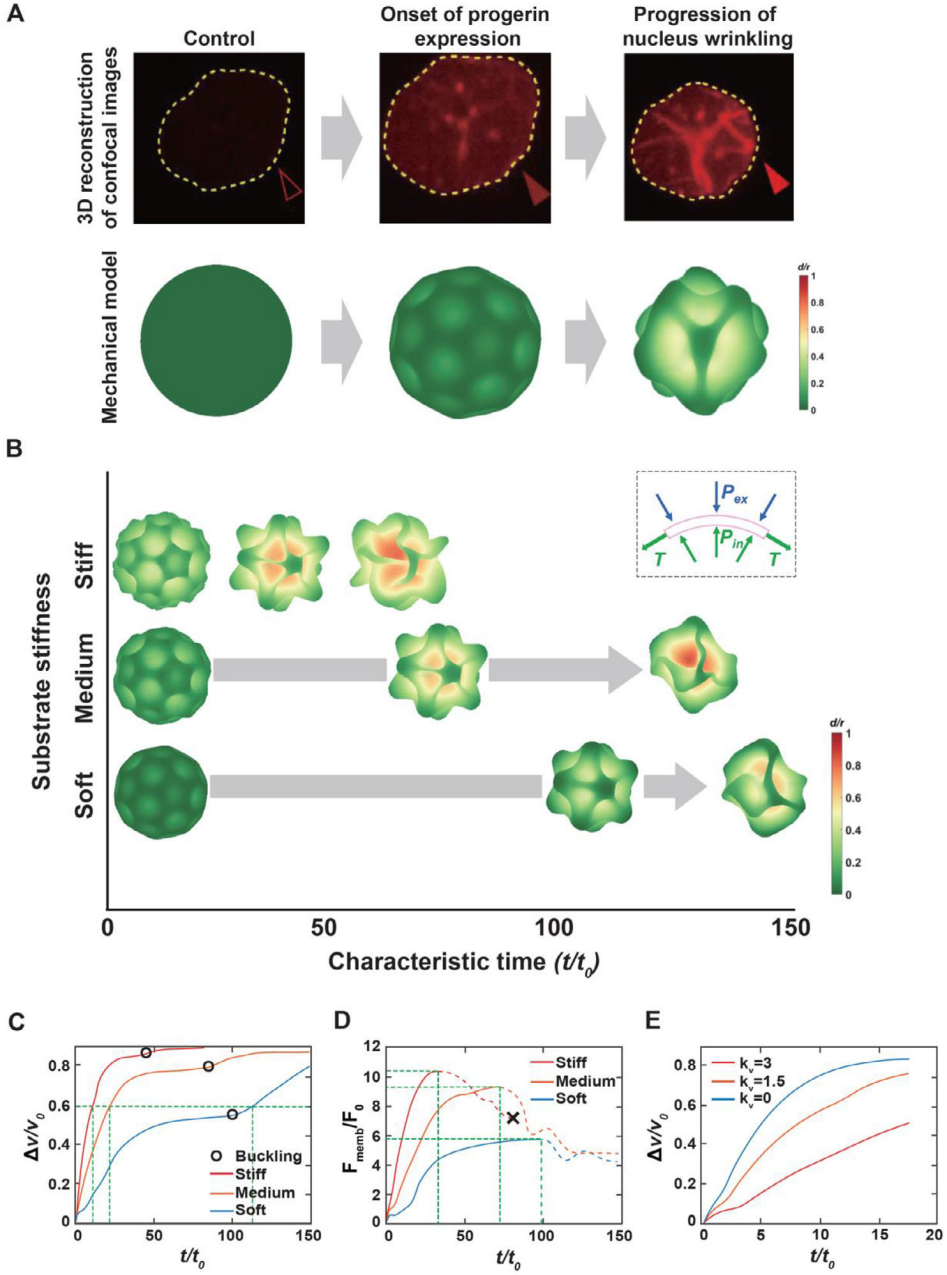
Mechanical model of nucleus wrinkling. A) Construction of a mechanical model mimicking progerin‐induced deformation of the nuclear surface. Experimental observations of Tet‐On‐inducible progerin expression are depicted by 3D reconstructions of confocal images (top) and the corresponding mechanical model (bottom), showing the smooth spherical shape of progerin‐absent control nuclei (left), a buckyball‐like surface configuration at the onset of progerin expression (middle), and a folded surface texture due to the progression of nuclear wrinkling (right). The color code represents displacement of the nuclear surface. B–E) Simulation of time‐dependent nucleus wrinkling in response to changes in substrate stiffness. Reduced pressure on the nucleus on compliant substrates delays surface wrinkling. The second‐order buckling (i.e., from a buckyball‐like pattern to a folded pattern) occurs at characteristic time scales of 45, 90, and 105 on stiff, medium, and soft substrates, respectively (B). (Inset) The local force balance in a membrane microelement, where membrane tension is mimicked by an equivalent internal pressure due to surface curvature and mechanical equilibrium. Time‐dependent volume changes of the nucleus on different stiffness substrates indicate that nuclei on soft substrates take the longest time, while those on stiff substrates take the shortest, based on the characteristic time to reach a specific volume change (C), where *∆V* and *V*_0_ represent the volume change and the initial volume, respectively, and black circles mark the accelerated collapses along the nucleus triggered by the second‐order buckling. The nuclear surface tension, calculated from nuclear volume change, shows that nuclei on stiff substrates have the highest tension value and largest change rate, while nuclei on soft substrates have the lowest tension value and smallest change rate (D), where the stretching force *F*_e_ was applied to characterize the membrane tension *F*_memb_, *F*_0_ represents the unit characteristic force, and the black cross indicates the breakdown of computational model due to the contact of the membrane under large deformation, respectively. Increasing internal pressure (i.e., enhanced membrane tension), corresponding to a greater 𝐾_𝑉_ value, indicating a stronger resistance to external pressure reduces nuclear volume change (E), indicating that reduced nuclear tension on soft substrates delays nuclear wrinkling compared to nuclei on stiff substrates.

To simulate NE wrinkling, a representative nuclear surface deformation induced by progerin expression, we developed a computational model by adapting nuclear geometry obtained from 3D‐reconstructed confocal images to establish a soft, spherical, elastic, thin shell as the initial model structure (**Figure** **3**A). The shell was discretized into triangular elements using a series of vertices to characterize the deformation of the nuclear surface. The potential energy generated by the movement of the vertices allows the shell to resist external forces (see the Experimental Section for details). Because the FRET analysis indicated reduced NE tension (tensile force) at the onset of progerin expression (Figure 2), we simplified the force transmitting from the external environment onto the nucleus, i.e., the cytoskeletal force, as a net pressure force. Furthermore, by combining our previous report demonstrating that the actin cytoskeleton exerts elevated pressure on the nucleus on a stiff substrate,^[^ ^40^ ^]^ we applied varying external pressures to model the nuclear response to changes in substrate stiffness, where pressure increases with increasing substrate stiffness.

The nuclear mechanical model revealed that shell wrinkling progresses in two stages: the smooth surface of the control nucleus first wrinkles into a buckyball‐like structure, followed by a transition from the buckyball‐like morphology to a labyrinthine pattern with deep invaginations along the nuclear surface (Figure 3A; Movie S3, Supporting Information). As substrate stiffness decreased, the buckyball‐to‐labyrinth transition was delayed at characteristic times (*t*/*t*_0_) of 45, 90, and 105 for stiff, medium, and soft substrates, respectively (Figure 3B), as measured by the relative volume change (Δ*v*/*v*_0_, Figure 3C). The nuclear membrane surface tension (*F*_memb_) quantitatively assesses the substrate‐stiffness‐dependent differential nuclear deformation, with the occurrence of the maximum value delayed by reducing substrate stiffness at *t*/*t*_0_ values of 30, 75, and 100 for stiff, medium, and soft substrates, respectively (Figure 3D). These results indicate that the shrinkage of the nuclear shell volume and the elevation of nuclear membrane tension are delayed by reducing substrate stiffness, consistent with our live‐cell monitoring, which showed that the nucleus on stiff substrates favors nuclear wrinkling in response to progerin expression (Figure 1). Since we used an internal pressure to generate membrane tension (inset, Figure 3B), a lower volume modulus (*K*_V_), corresponding to a smaller ability to resist volume change, induces lower membrane tension. This low membrane tension, i.e., low internal pressure, accelerated nuclear wrinkling (Figure 3E; Movie S3, Supporting Information), confirms that a greater net external pressure leads to faster and deeper wrinkling.

These results demonstrate that reduced nuclear membrane tension in cells placed on soft substrates delays the occurrence of nuclear wrinkling. They further suggest that nuclear wrinkling, induced by the localized accumulation of permanently farnesylated prelamin A along the nuclear membrane, could disrupt the nuclear–cytoskeletal connection that is highly sensitive to changes in substrate stiffness.

### 2.4 Mechanosensitive Progerin Expression Induces Temporal Alterations in Gene Profiling

Nuclear deformation is not merely a phenotypic signature of disease but is strongly indicative of nuclear force‐dependent alteration of genome architecture and dysregulation of gene expression.^[^ ^41^ ^]^ Since progerin‐induced nuclear wrinkling is attributed to reduced NE tension and this biochemical response is tightly regulated by mechanosensing of substrate stiffness (Figures 1, 2, 3), we investigated whether progerin‐induced temporal differential NE wrinkling could also alter gene expression profiles in response to changes in substrate stiffness.

**Figure 4:**
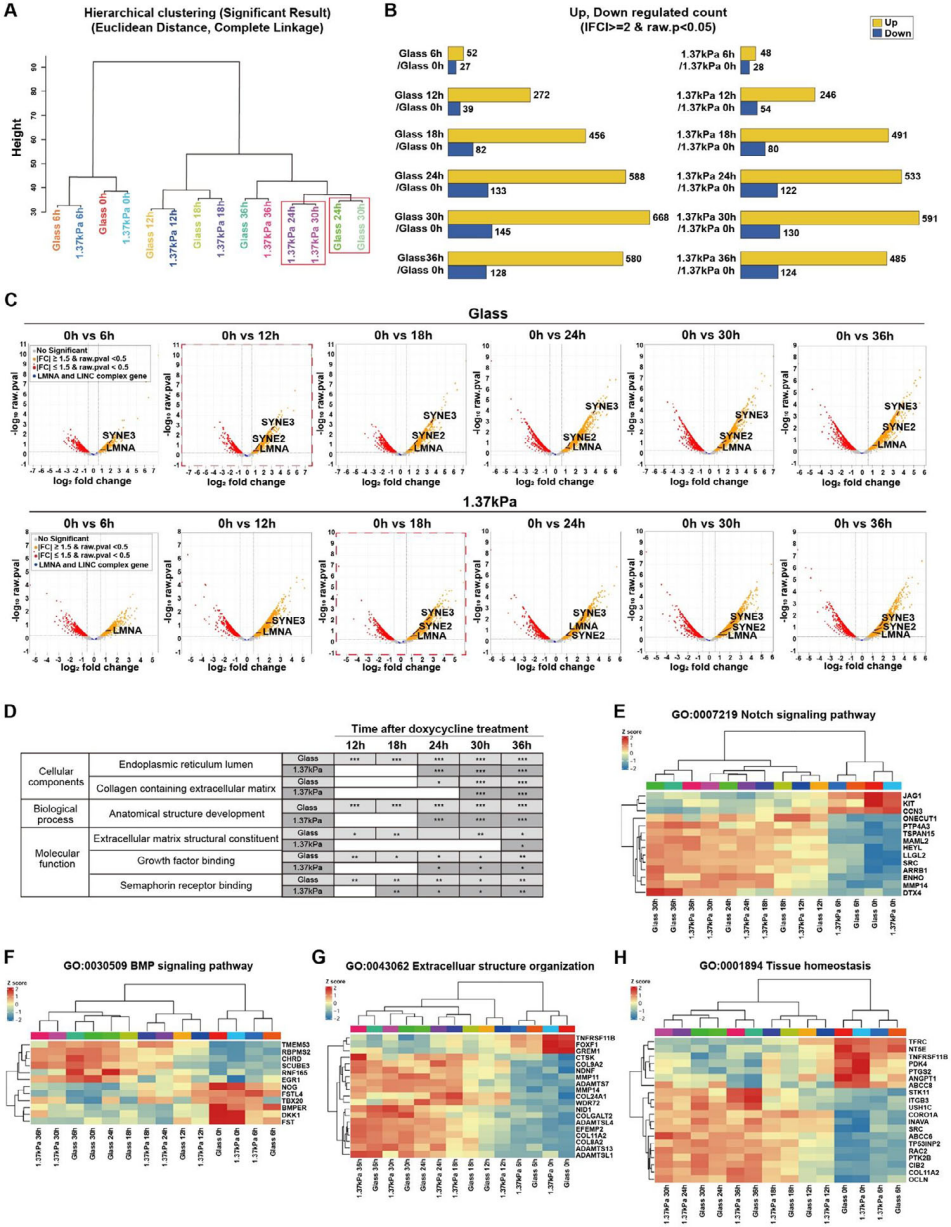
Time‐dependent alteration of gene profiling and signaling pathways in response to mechanosensitive progerin expression. A–D) Progerin‐induced, time‐dependent differential gene expression in response to changes in substrate stiffness (stiff glass substrates vs soft PAG substrates of 1.37 kPa). The similarity in gene expression profiles was assessed by Euclidean distance and complete linkage clustering, where the height of the dendrogram represents the Euclidean distance between clusters, indicating the similarity in expression profiles (A). Note that gene expression profiles remained similar between cells on stiff glass and soft PAG substrates at 18 h but clustered by substrate stiffness at 24 and 30 h (marked by red boxes), and reclustered at 36 h. The number of differentially regulated genes is displayed for each substrate stiffness by comparing with the doxycycline‐untreated control condition (glass 0 h on the left, 1.37 kPa 0 h on the right), where yellow and blue bars indicate upregulated and downregulated genes, respectively (B). Volcano plots display doxycycline treatment time‐dependent evolution of log2 fold changes in LMNA and LINC complex‐associated genes (e.g., *SUN1*, *SUN2*, *SYNE1*, *SYNE2*, and *SYNE3*) on stiff glass substrates (top row) or soft PAG substrates (bottom row) (C). Note that while LMNA expression increased from 6 h after doxycycline treatment on both stiff glass and soft PAG substrates, significant increases in *LMNA*, *SYNE2*, and *SYNE3* were observed at 12 h on stiff substrates but at 18 h on soft substrates (marked by red dotted boxes), indicating delayed expression of nesprin on soft substrates. Gene ontology (GO) analysis was performed comparing the control glass and soft PAG substrates at different doxycycline treatment times (12, 18, 24, 30, and 36 h) for cellular components, biological processes, and molecular functions, with term sizes between 10 and 500 (D). In panel B, the criteria for significant changes in gene expression were fold change ≥ |2| and raw *p*‐value < 0.05. In panel C, yellow and red dots indicate specific gene expression levels corresponding to fold change ≥ |1.5|, raw *p*‐value < 0.5, and fold change ≤ |1.5| with raw *p*‐value < 0.5, respectively, with LINC complex‐associated genes colored blue. In panel D, adjusted *p*‐values reported from g:Profiler were derived using a one‐sided hypergeometric test and corrected by the Benjamini–Hochberg method (***: *p* < 0.001, **: *p* < 0.01, *: *p* < 0.05). E–H) Heatmap analysis of GO terms related to mechanosensing of substrate stiffness. Representative signaling pathways, including the Notch signaling pathway (GO:0007219, E), BMP signaling pathway (GO:0030509, F), extracellular structure organization (GO:0043062, G), and tissue homeostasis (GO:0001894, H), were visualized. Euclidean distance was used as the distance metric, and complete linkage was applied for hierarchical clustering in the analysis of each dataset. For further details, refer to the Experimental Section.

To systematically analyze gene expression over time, we performed RNA sequencing for cells cultured on either a stiff glass substrate or a soft PAG substrate at 1.37 kPa, followed by doxycycline treatment at 6 h intervals for up to 36 h (**Figure** **4**). Hierarchical clustering of gene expression, determined by pairwise complete‐linkage clustering analysis, revealed that the tested samples were primarily grouped by doxycycline treatment time, irrespective of substrate stiffness (i.e., 0, 6, 12, 18, and 36 h). However, only the samples treated with doxycycline for 24 and 30 h formed distinct groups based on substrate stiffness, i.e., soft 1.37 kPa substrates at 24 and 30 h versus stiff glass substrates at the same time points (Figure 4A; Figure S5A, Supporting Information). Further analysis of the number of differentially expressed genes (DEGs) in experimental conditions compared to the doxycycline‐untreated control group (denoted as glass 0 h or 1.37 kPa 0 h) indicated that the largest changes in gene expression levels, including both upregulation and downregulation, occurred at 24 and 30 h after doxycycline treatment for each substrate stiffness (Figure 4B). Interestingly, these time points coincided with the reduction of NE tension and the formation of nuclear wrinkles, occurring at 24 and 25 h on stiff substrates, or 30 and 32 h on soft substrates, respectively (Figures 1 and 2). These results imply that mechanosensitive progerin expression leads to differential changes in gene expression as NE tension‐dependent nuclear wrinkling progresses.

Since the LINC complex bridging between the extra‐nuclear–cytoskeletal network and nuclear membrane‐associated proteins that physically interact with chromosomal architecture regulates the transmission of biophysical stimuli into the nucleus,^[^ ^21^, ^41^ ^]^ we conducted RNA sequencing to investigate whether progerin expression regulates the expression of LINC complex‐associated genes encoding SUN and nesprin proteins. Compared to the doxycycline‐untreated control conditions, our results showed a significant increase in the expression of *LMNA*, *SYNE2*, and *SYNE3* genes in response to increasing doxycycline treatment across substrate stiffness, where we also observed doxycycline treatment time‐dependent differential expression of these genes (Figure 4C). For instance, *LMNA* gene expression was observed at 6 h in both stiff and soft substrates, whereas *SYNE2* and *SYNE3* expression increased after 12 h of doxycycline treatment on stiff substrates but after 18 h on soft substrates (Figure 4C). These findings align with previous results showing that doxycycline‐induced progerin expression (Figure 1), reduction of nuclear tension (Figure 2), and nuclear wrinkling (Figures 1, 2, 3) were delayed on soft substrates compared to cells on stiff substrates.

To further investigate whether substrate stiffness‐dependent differential gene expression induced by doxycycline‐inducible progerin expression could functionally alter mechanosensation‐mediated pathways, we used the gene set enrichment analysis (GSEA) database (Figure S5B–E, Supporting Information). Specifically focusing on actomyosin‐related genes (GSEA C5>GO, 286 genes) and nuclear membrane‐related genes (GSEA C5>GO, 417 genes) obtained after 24 h of doxycycline treatment, 13 actomyosin‐related genes and 17 nuclear membrane‐related genes were identified as DEGs (Figure S5B–D, Supporting Information). Furthermore, the changes in the expression of these DEGs occurred 24 h after doxycycline treatment on stiff substrates and 30 h after doxycycline treatment on soft substrates (Figure S5E, Supporting Information).

Among the top 20 GO enrichment analysis results, based on adjusted *p*‐values, our investigation of cellular components, biological processes, and molecular functions identified a significant delay in soft substrates compared to that in stiff substrates (Figure 4D). These results were compared with RNA sequencing data from the gene expression omnibus public datasets GSE141950 and GSE118633, which analyzed dermal fibroblasts from healthy individuals and patients with HGPS (Figure S6, Supporting Information). GSE141950 analysis revealed distinct gene expression patterns in fibroblasts from patients with HGPS (Figure 6A,B, Supporting Information). Furthermore, pathway enrichment analysis of cellular components, biological processes, and molecular functions revealed similar changes in these pathways (Figure 4D; Figure S6C, Supporting Information). Moreover, GSE118633 analysis revealed distinct gene expression patterns in fibroblasts from patients with HGPS, confirming changes in the same pathways (Figure 4D; Figure S6D–F, Supporting Information). These results not only confirm that our developed Tet‐On‐inducible progerin‐expressing HeLa cells effectively model HGPS but also demonstrate that substrate stiffness‐dependent differential progerin expression could modify the onset of gene expression that regulates multiple signaling pathways.

Finally, we evaluated whether mechanosensitive progerin expression could regulate Notch signaling and bone morphogenetic protein (BMP) signaling. The Notch signaling pathway, highly sensitive to mechanical signals, regulates cell and tissue fate in most tissues,^[^ ^42^ ^]^ and the BMP signaling pathway, involving nuclear membrane proteins, is directly regulated by mechanical signal transduction pathways without autocrine ligands, occurring at the receptor, cytoplasmic, and nuclear levels.^[^ ^43^ ^]^ Heatmap analysis confirmed that Notch and BMP signaling were altered by doxycycline‐induced progerin expression in a substrate stiffness‐dependent manner (Figure 4E,F). Furthermore, we observed a distinct regulation of gene expression involved in extracellular structure organization in response to changes in substrate stiffness (Figure 4G), which is necessary for tissue homeostasis.^[^ ^44^ ^]^ Heatmap analysis of tissue homeostasis‐regulating differential gene expression further confirmed that mechanosensitive progerin expression could functionally regulate tissue homeostasis (Figure 4H).

Together, these results suggest that substrate stiffness‐dependent differential progerin expression regulates gene expression, ultimately altering mechanosensitive signaling pathways.

### 2.5 Mechanosensitive Progerin Expression Modulates the Spatiotemporal Reorganization of Heterochromatin

Progerin exhibits a strong binding affinity for histone‐lysine *N*‐methyltransferase SUV39H1, preventing its proteasomal degradation and thereby increasing epigenetic modifications to the DNA packaging protein histones, e.g., H3K9me3. This, in turn, reduces DNA repair capacity and accelerates senescence.^[^ ^45^ ^]^ Meanwhile, elevated substrate stiffness correlates with enhanced levels of H3K9me2/3.^[^ ^46^ ^]^ Thus, we investigated whether the substrate stiffness‐dependent mechanosensitive alteration of progerin expression could modulate H3K9me2/3 levels, indicative of heterochromatin formation and transcriptional silencing.

**Figure 5:**
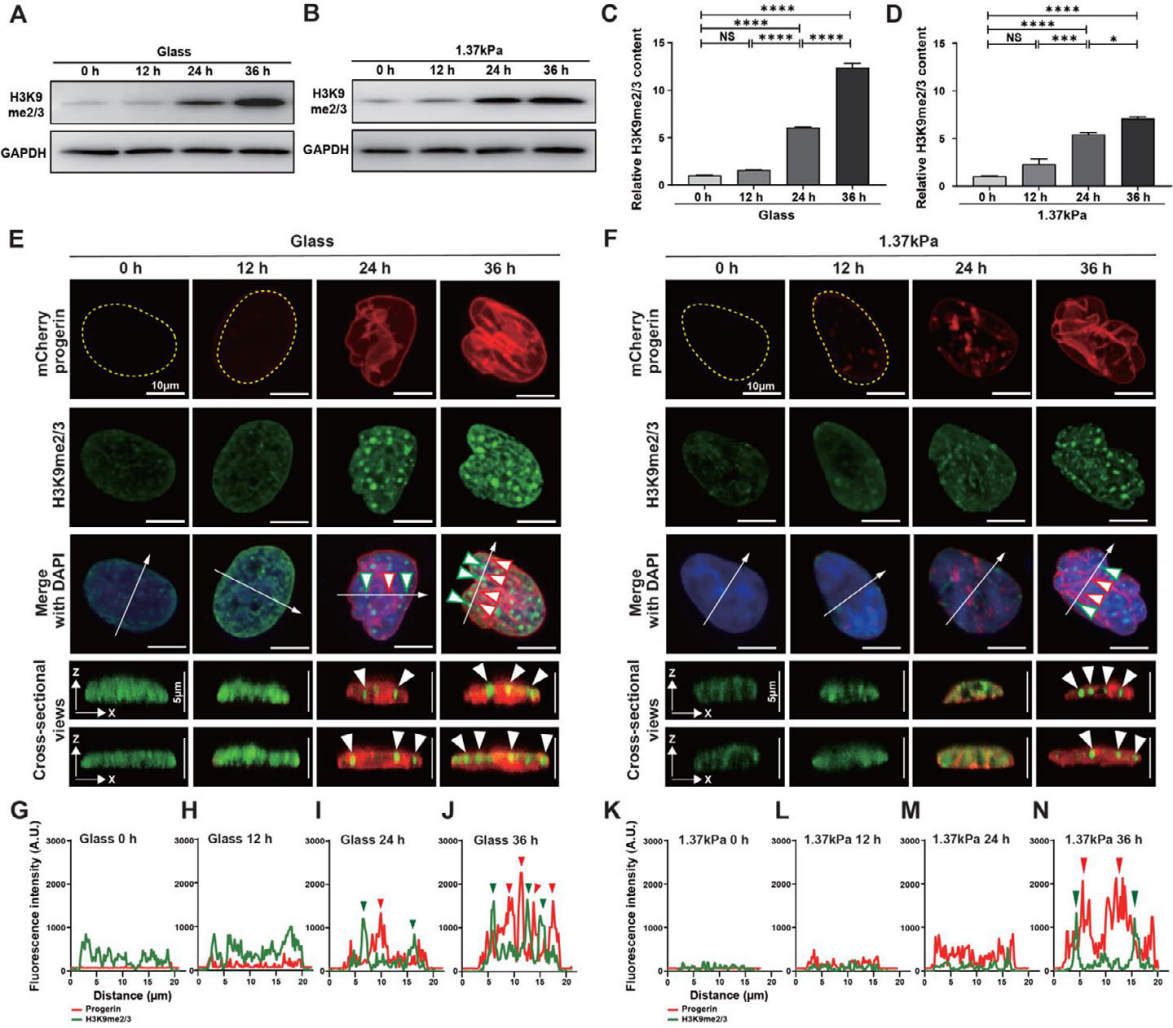
Time‐dependent differential epigenetic modifications in response to mechanosensitive progerin expression. A–D) Quantification of doxycycline treatment time‐dependent differential expression of H3K9me2/3 in response to changes in substrate stiffness. H3K9me2/3 expression was quantified by immunoblotting against H3K9me2/3 and GAPDH antibodies in progerin‐expressing Tet‐On HeLa cells placed on control stiff substrates (glass, A) and soft PAG substrates (1.37 kPa, B) with doxycycline treatment every 12 h for up to 36 h. (C,D) Total protein expression increased with doxycycline treatment in each condition. In panels C and D, three independently performed experiments were averaged and normalized to the values in 0 h condition. Error bars indicate the S.E.M., and one‐way ANOVA using Tukey's test was applied for comparison between groups (****: *p* < 0.0001, ***: *p* < 0.005, *: *p* < 0.05, NS: not significant). E–N) Spatiotemporal alterations of heterochromatic histone modifications in doxycycline‐induced progerin‐expressing cells placed on varying substrate stiffness. Tet‐On HeLa cells expressing mCherry‐tagged Δ50 LMNA (red) placed on control glass (E) and PAG substrates of 1.37 kPa (F) were immunostained for nuclei (DAPI, blue) and H3K9me2/3 (green) every 12 h after doxycycline treatment. 3D‐rendered nuclei, reconstructed from z‐stacked confocal fluorescent images, depict that doxycycline treatment time‐dependently increased progerin expression, inducing H3K9me2/3 clustering in the nuclear interior (E–N). H3K9me2/3 clusters were detected after 24 h on stiff substrates (glass, E,G–J) but appeared after 36 h on soft substrates (1.37 kPa, F,K–N). Yellow dotted lines indicate the nuclear boundary as determined by DAPI staining; white arrowheads indicate clustered H3K9me2/3 (E,F). Fluorescence intensity profiles monitored by line scanning through the maximum intensity projected nuclear images show that H3K9me2/3 clusters largely alternate with progerin staining. More intensive peaks were detected in nuclei of cells placed on stiff substrates compared to those on soft substrates (G–J vs K–N), where red and green arrowheads indicate fluorescence intensity peaks corresponding to progerin and H3K9me2/3 expression, respectively.

To assess H3K9me2/3 protein expression levels, western blot analysis was performed on cells cultured on either stiff glass substrates or soft PAG substrates (1.37 kPa), which were treated with doxycycline at 12 h intervals for up to 36 h (**Figure** **5**A–D). Cells cultured on stiff glass substrates showed a significant increase in H3K9me2/3 expression 24 h after doxycycline treatment, with levels doubling by 36 h (Figure 5A,C). In contrast, cells cultured on soft PAG substrates showed increased H3K9me2/3 expression after 24 h of treatment, but the levels only slightly increased further by 36 h (Figure 5B,D). These results suggest that temporal alteration of histone modifications is also accompanied by the substrate stiffness‐dependent differential progerin expression.

Given that spatially resolved epigenetic modifications of histones can induce specific alterations in chromatin accessibility and transcriptional phenotypes,^[^ ^46^ ^]^ we also investigated the intranuclear distribution of H3K9me2/3 (Figure 5E–N). Cross‐sectional analysis of 3D‐reconstructed confocal images of nuclei costained for progerin and H3K9me2/3 revealed that increased H3K9me2/3 levels were associated with clustering of H3K9me2/3 within the nucleus 24 h after doxycycline treatment on stiff substrates, where H3K9me2/3 clustering was alternatively stained for progerin expression (Figure 5E). Fluorescence intensity profiles obtained by line scanning through the nuclear interior confirmed that progerin expression and H3K9me2/3 clustering displayed distinct alternative staining patterns at 24 h, with fluorescence profiles intensifying by 36 h (Figure 5G–J). However, on soft PAG substrates, intranuclear H3K9me2/3 clustering predominated after 36 h of doxycycline treatment, coinciding with enhanced progerin expression at the same time (Figure 5F). Line scanning through the nuclear interior revealed distinct alternative staining for H3K9me2/3 clusters and progerin at 36 h (Figure 5K–N).

These results indicate that substrate stiffness‐dependent differential onset of progerin expression leads to a time‐dependent progression of heterochromatin levels, accompanied by clustering of H3K9me2/3 within the nucleus. This strongly suggests that the delayed gene expression in cells placed on soft substrates, which transmit reduced forces to the nucleus, can be attributed to incomplete heterochromatin structure.

### 2.6 Substrate Stiffness‐Dependent Differential Progerin Expression Is Mediated by Distinct Transcription Factor Binding Motifs

Alterations in gene expression and epigenetic modifications are regulated by substrate stiffness‐dependent differential progerin expression (Figures 4 and 5). In line with previous reports indicating that lamin A depletion enhances chromatin mobility,^[^ ^47^ ^]^ these results strongly imply that progerin‐induced differential gene expression patterns in response to changes in substrate stiffness can be attributed to variations in transcription factor binding motifs.

**Figure 6:**
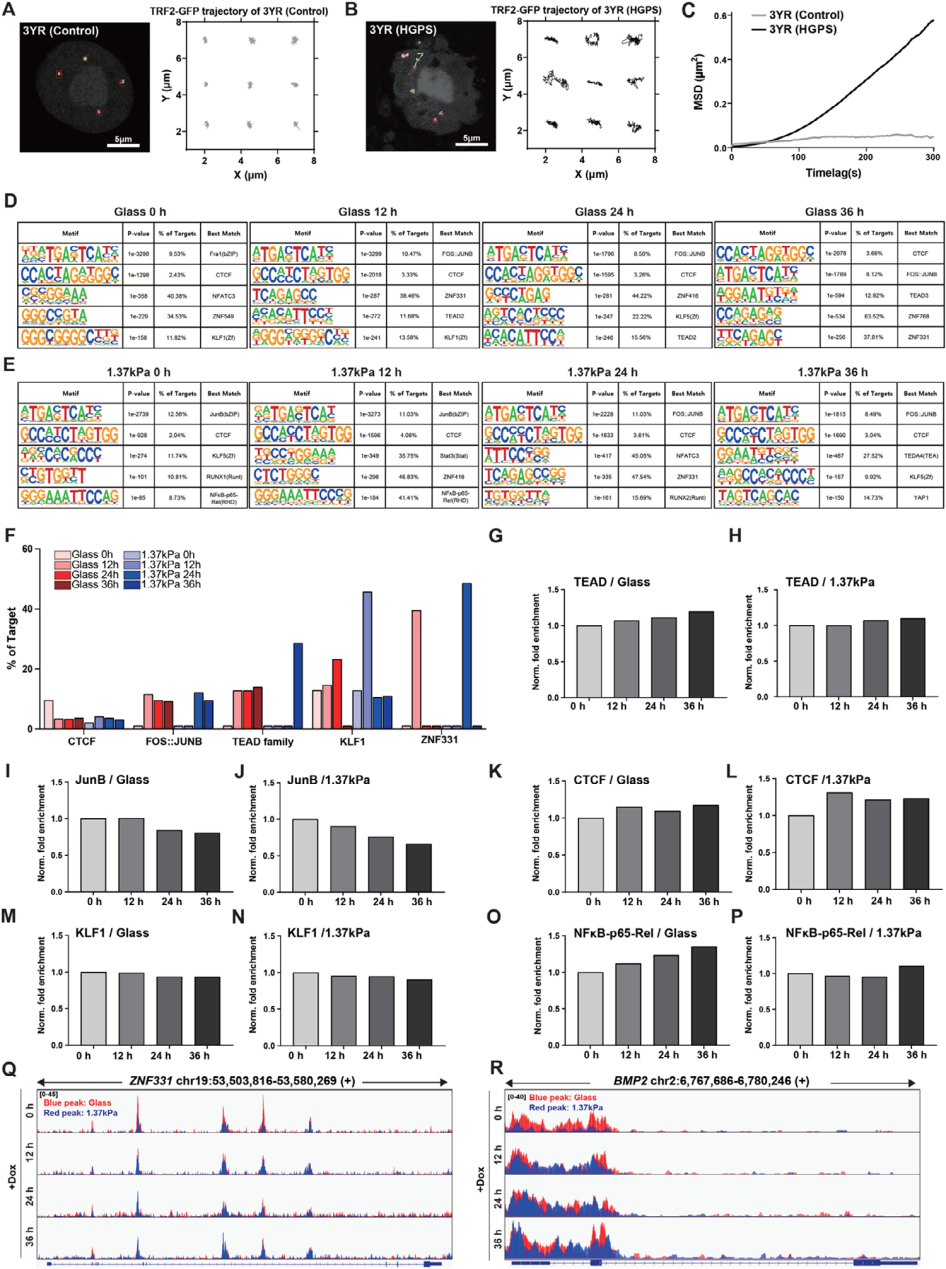
Differential chromatin accessibility in response to mechanosensitive progerin expression. A–C) Differential chromatin mobility in response to progerin expression. Time‐lapse tracking of fluorescence‐tagged chromatin was performed in TRF2 (telomeric repeat‐binding factor 2)‐transfected human dermal fibroblasts obtained from a three‐year‐old healthy control (denoted as 3 YR (Control), A) and an HGPS patient (denoted as 3 YR (HGPS), B), where nine randomly selected chromatin trajectories are displayed. (C) Quantitative analysis of the mean squared displacement (MSD) at each time lag indicates enhanced chromatin mobility in HGPS patients compared to the healthy control. D–R) ATAC sequencing‐based identification of differential key transcription factor (TF) binding motifs in response to substrate stiffness‐dependent progerin expression. Representative de novo TF binding motifs in Tet‐On HeLa cells expressing progerin were identified between cells on control stiff substrates (denoted as glass, D) and cells on soft PAG substrates (denoted as 1.37 kPa, E) at 12 h intervals after doxycycline treatment for 36 h using Homer software. The bar graph indicates the percentage of target binding motifs for *CTCF*, *FOS::JUNB*, *TEAD* family, *KLF1*, and *ZNF331* (F), where each bar represents fold enrichment, defined as the percentage of target sequences with the motif divided by the percentage of background sequences with the motif. Data are normalized to doxycycline‐untreated control groups (denoted as glass 0 h, 1.37 kPa 0 h). Doxycycline treatment increased fold enrichment for *TEAD* (G,H), *CTCF* (K,L), and *NFkB‐p65‐Rel* (O,P), but decreased fold enrichment for *JunB* (I,J) and *KLF1* (M,N) in control stiff substrates, with these changes diminished in soft PAG substrates. ATAC‐seq tracking of *ZNF331* (Q), a known transcriptional repressor, and *BMP2* (R), a component of mechanosensory pathways, was visualized using Integrative Genomics Viewer (IGV), where blue and red peaks indicate stiff glass and soft PAG substrates, respectively.

To test this hypothesis, we first examined whether progerin expression alters internuclear chromatin dynamics by comparing telomere motion in dermal fibroblasts derived from a 3‐year‐old healthy individual and a same‐aged patient with HGPS, denoted as 3YR (control) and 3YR (HGPS), respectively (**Figure** **6**A–C). Time‐lapse monitoring of GFP‐labeled telomeres bound by the telomeric repeat‐binding factor 2 (TRF2) revealed enhanced telomere movement in HGPS cells compared to control cells (Figure 6A,B). Single‐particle tracking, followed by calculation of the mean squared displacement (MSD) for the recorded trajectories, confirmed more diffusible chromatin motion in HGPS cells than in control cells, representing a confined motion (Figure 6C; Movie S4, Supporting Information).

Because these results strongly imply that nuclear wrinkling, typically observed in patients with HGPS, is associated with enhanced chromatin dynamics due to progerin‐induced loss of mechanical integrity along the nuclear lamina,^[^ ^47^, ^48^ ^]^ we further investigated whether progerin‐induced differential chromatin dynamics regulate genome‐wide chromatin accessibility. Specifically, we performed a high‐throughput assay for transposase‐accessible chromatin using sequencing (ATAC‐seq) to assess whether progerin‐induced distinct, time‐dependent gene expression patterns in response to changes in substrate stiffness are attributable to variations in transcription factor binding motifs. The calculation of transcription start site (TSS) enrichment scores revealed that chromatin‐accessible regions are enriched at TSSs, and the distribution of aligned fragment lengths obtained from all tested samples confirmed the high quality of ATAC‐seq data (Figure S7A,B, Supporting Information). We observed that chromatin‐accessible regions were similarly distributed across the genome under different experimental conditions (Figure S7C,D, Supporting Information). Analysis of transcription factor (TF) binding motifs within chromatin‐accessible regions revealed specific changes in TF binding motifs in response to doxycycline‐induced progerin expression on stiff glass substrates (denoted as glass, Figure 6D) and soft PAG substrates (denoted as 1.37 kPa, Figure 6E), selected from the top ten TFs predicted to have binding motifs in chromatin‐accessible regions for each condition (Figure S7E,F, Supporting Information).

In particular, as represented by the percentage of targets, we noted that the expression of differential TF‐binding motifs for CCCTC‐binding factor (*CTCF*), *FOS::JUNB*, TEA domain (*TEAD*) family, Kruppel‐like factor 1 (*KLF1*), and zinc finger protein 331 (*ZNF331*) was delayed on soft substrates compared to stiff substrates (Figure 6F). These results suggest that progerin‐induced differential gene expression (Figure 4), following enhanced H3K9me2/3 clustering (Figure 5), could be attributed to differences in TF‐binding motifs. Progerin‐induced alterations in heterochromatin structure could modulate the configuration of TF binding, ultimately regulating substrate stiffness‐dependent gene expression.

A systematic comparison of fold enrichment values normalized to the untreated doxycycline group (0 h) identified differentially regulated TFs that promote multiple aging‐associated cellular mechanisms, while all the detected TFs were expressed at lower levels on soft substrates than on stiff substrates (glass vs 1.37 kPa, Figure 6G–P). For instance, the expression of *TEAD* (Figure 6G,H), *CTCF* (Figure 6K,L), and *NFkB‐p65‐Rel* (Figure 6O,P), regulating pathological processes in HGPS, including cell proliferation, chromatin structure, and inflammatory responses,^[^ ^49^ ^]^ progressively increased with doxycycline treatment. In contrast, the expression of *JunB* (Figure 6I,J) and *KLF1* (Figure 6M,N), regulating cellular stress responses (e.g., oxidative stress and inflammation)^[^ ^50^ ^]^ and gene transcription in erythroid differentiation,^[^ ^51^ ^]^ respectively, gradually decreased with doxycycline treatment. Meanwhile, mechanosensitive signaling‐mediated *ZNF331*, a transcriptional repressor containing the Kruppel‐associated box (KRAB) domain,^[^ ^52^ ^]^ exhibited an increase in ATAC‐seq peak height following doxycycline treatment, indicating enhanced accessibility of the *ZNF331* gene (Figure 6Q). In contrast, the gene accessibility of *BMP2*, involved in the BMP signaling pathway, one of the key pathways identified in mechanosensation (Figure 4F), increased progressively by doxycycline‐induced progerin expression (Figure 6R).

These findings suggest that nuclear wrinkling induced by progerin expression elevates chromatin dynamics, enhances heterochromatin clustering, and ultimately regulates chromatin accessibility.

### 2.7 Progerin‐Induced Nuclear Deformation Is Mediated by the Remodeling of LINC Complex‐Dependent Molecular Connections with LMNA

Lamin A interacts with the cytoskeleton via the LINC complex, which is composed of SUN proteins in the inner nuclear membrane and nesprin isoforms, the cytoplasmic domains of KASH proteins in the outer nuclear membrane, enabling the transmission of cytoskeletal forces to the nuclear membrane.^[^ ^53^ ^]^ We previously demonstrated that progerin expression reduced nuclear tension by incorporating the nesprin tension sensor, mimicking nesprin 2 bridging between the actin cytoskeleton and SUN proteins (Figure 2). Based on this, we hypothesized that progerin‐induced nuclear deformation could be mediated by the differential interactions between the LINC complex and LMNA.

**Figure 7:**
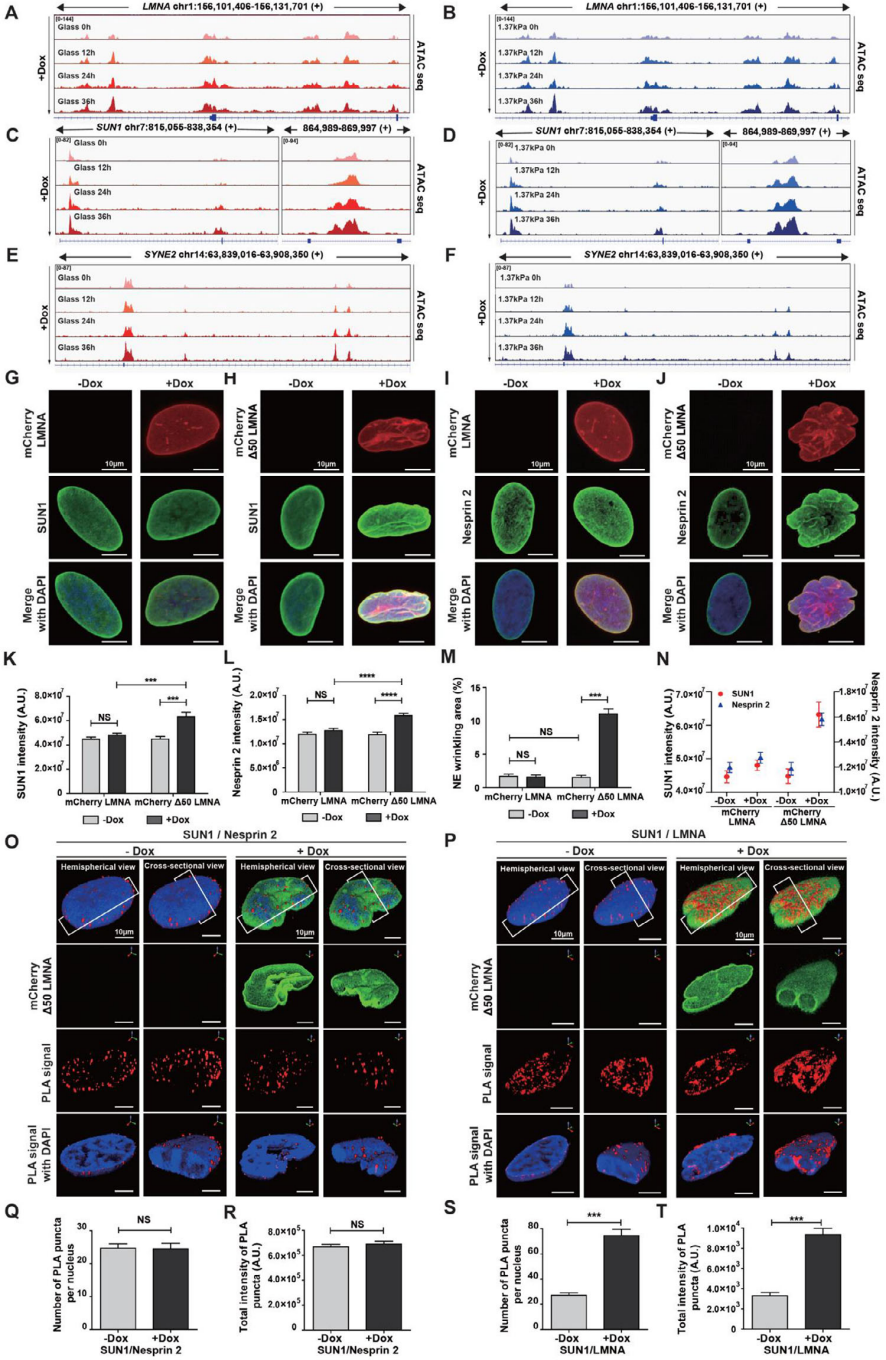
LINC complex‐mediated remodeling of LMNA‐associated nuclear tethering in progerin‐expressing cells. A–F) ATAC‐seq‐based identification of chromatin accessibility for LMNA‐associated LINC complex components in progerin‐expressing cells. ATAC‐seq peaks for *LMNA* (A,B), *SUN1* (C,D), and *SYNE2* (E,F) in progerin expression‐induced cells treated with doxycycline at 12 h intervals for up to 36 h on control glass (A,C,E) and soft PAG substrates (B,D,F) were visualized by Integrative Genomics Viewer (IGV). Note that as doxycycline treatment progresses, peaks for *LMNA*, *SUN1*, and *SYNE2* in both control glass and soft PAG increase, indicating enhanced chromatin accessibility, but the peak height in soft substrates remains lower than that in control glass, approaching a similar level at 36 h. G–N) Differential expression of NE‐associated proteins and nuclear wrinkling in response to progerin expression. Tet‐On HeLa cells expressing mCherry‐tagged LMNA or Δ50 LMNA (progerin) were immunostained for SUN1 (green) and nucleus (DAPI, blue) (G,H) or nesprin 2 (green) and nucleus (DAPI, blue) (I,J) before (−Dox) and after (+Dox) doxycycline treatment. While doxycycline‐induced expression of mCherry‐tagged LMNA did not alter the SUN1 (G,K) and nesprin 2 contents (I,L), doxycycline‐induced expression of mCherry‐tagged Δ50 LMNA significantly increased SUN1 (H,K) and nesprin 2 (J,L). Compared to doxycycline‐induced LMNA expression, which did not induce changes in nuclear shape (G,I,M), Δ50 LMNA expression significantly increased NE wrinkling (H,J,M). SUN1 and nesprin 2 expression was more sensitive to Δ50 LMNA expression than to LMNA expression (N). In panels K–N, >50 nuclei were analyzed for each condition; error bars indicate the standard error of the mean (S.E.M.); and Student's *t*‐test was applied for comparison between two groups (****: *p* < 0.0001, ***: *p* < 0.001, NS: not significant). O–T) Progerin‐induced differential interaction in LMNA‐associated LINC proteins. The strength of molecular interaction between SUN1 and nesprin 2 (O,Q,R) or between SUN1 and LMNA (P,S,T) was estimated by quantifying the number and total intensity of proximity ligation assay (PLA) signals (red dots) in DAPI‐stained nuclei (blue). Hemispherical and cross‐sectional views of 3D‐rendered nuclei showed that punctate PLA signals were preferentially localized along the nuclear periphery. Note that doxycycline‐induced progerin expression significantly increased the PLA signals of SUN1 associated with LMNA (S,T), while PLA signals of SUN1 associated with nesprin 2 remained unchanged (Q,R). In panels Q, R, S, and T, >50 nuclei were analyzed for each condition; error bars indicate the S.E.M.; and an unpaired *t*‐test was applied to assess statistical significance (***: *p* < 0.001, NS: not significant).

To determine whether the evolution of NE wrinkling in response to progerin expression was associated with chromosomal interactions between DNA and LINC proteins, we first analyzed chromatin accessibility of *LMNA*, *SUN1*, and *SYNE2* using ATAC‐seq (Figure S8A–F, Supporting Information). After doxycycline treatment, these genes progressively enhanced chromatin accessibility on both stiff and soft substrates (**Figure** **7**A–F). While *LMNA* gene accessibility increased on both substrate types 12 h post‐doxycycline treatment, however, the accessibility of *SUN1* and *SYNE2* genes was delayed on soft substrates, with a similar increase observed on both stiff and soft substrates at 36 h (Figure 7A–F; Figure S8A–F, Supporting Information). Consistent with hierarchical clustering analysis of RNA‐seq indicating that samples were clustered by substrate stiffness at 24 and 30 h, but clustered by doxycycline treatment time at 36 h (Figure 4A), these results suggest that substrate stiffness‐dependent differential chromatin accessibility was dominant at 24 h of progerin expression, but diminished at 36 h of doxycycline exposure. Together with the previous result exhibiting a delayed reduction of NE tension on soft substrates (Figure 2), these findings suggest that Tet‐On‐inducible progerin expression modulates *LMNA* gene expression, simultaneously upregulating *SUN1* and *SYNE2*, while reduced NE tension induced by progerin expression facilitates LINC complex‐mediated molecular binding.

To test this notion, we examined the expression levels of SUN1 and its binding partner, nesprin 2, in response to progerin expression by comparing mCherry‐tagged LMNA and progerin (denoted as Δ50 LMNA)‐expressing Tet‐On HeLa cells (Figure 7G–J). Quantitative immunofluorescence microscopy revealed that both SUN1 and nesprin 2 were upregulated in progerin‐expressing cells but not in *LMNA*‐overexpressing cells (Figure 7K,L), which was confirmed by immunoblotting analysis of total protein levels (Figure S8G–J, Supporting Information). These results demonstrate that the upregulation of LINC proteins is specifically induced by progerin expression and not by the accumulation of intact LMNA. To assess whether LINC complex proteins regulate progerin‐induced nuclear deformation, we compared changes in NE wrinkling before and after doxycycline‐induced expression of LMNA or progerin. As expected, NE wrinkling remained unchanged before doxycycline treatment and was not altered by *LMNA* expression (left two bars, Figure 7M). However, progerin expression resulted in a sixfold increase in NE wrinkling (right two bars, Figure 7M). Moreover, we confirmed that LINC complex proteins were more tightly regulated by progerin expression than by intact *LMNA* expression, i.e., the expression levels of *SUN1* and nesprin 2 increased proportionally with progerin expression but not with *LMNA* expression (Figure 7N). These results support the association between NE wrinkling and the upregulation of LINC complex proteins in response to progerin expression.

Building on previous reports that *SUN1* and *SUN2* are not functionally equivalent for nuclear connection to the actin cytoskeleton,^[^ ^54^ ^]^ and that SUN1, but not SUN2, is upregulated in HGPS cells,^[^ ^28^ ^]^ we investigated whether doxycycline‐activated progerin expression could remodel the molecular connectivity of LINC‐associated proteins to the nucleus via upregulation of *SUN1*. We assessed whether *SUN1* overexpression altered *LMNA*‐associated molecular interactions in the nuclear lamina, leading to progerin‐mediated nuclear deformation. To quantitatively analyze the interaction between *SUN1* and *LMNA* or nesprin 2 in response to progerin expression, a proximity ligation assay (PLA) was performed (Figure 7O–T). As expected, a majority of punctate PLA signals were preferentially localized along the nuclear periphery, where LINC components form a molecular assembly with lamin proteins (Figure 7O,P). By counting the number and total intensity of fluorescent dots representing molecular interactions, we observed that doxycycline‐induced progerin expression significantly increased the association between SUN1 and *LMNA* (Figure 7P,S,T), whereas the interaction between *SUN1* and nesprin 2 remained unchanged (Figure 7O,Q,R).

Together with the upregulation of *SUN1* in progerin‐expressing cells, the preservation of identical molecular interactions between *SUN1* and nesprin 2 further implies that progerin accumulation in intact *LMNA* enhances the spatial proximity of *SUN1* to *LMNA*, strongly suggesting that progerin expression induces nuclear deformation through the remodeling of *SUN1*‐dependent molecular connections with *LMNA*.

### 2.8 Actomyosin Contractility Regulates Nuclear Deformation by Altering Nuclear Tension in Progerin‐Expressing Cells

We previously demonstrated that progerin‐induced NE wrinkling was highly mechanosensitive (Figure 1) and that substrate stiffness‐dependent cytoskeletal tension mediated nuclear force at the nucleus–cytoskeletal interface (Figure 2). In conjunction with recent studies showing increased F‐actin polymerization in LMNA‐depleted human retinal pigment epithelial cells^[^ ^29^ ^]^ and elevated RhoA activation in Z24^−/−^ MSCs,^[^ ^30^ ^]^ we hypothesized that progerin‐induced nuclear deformation could be regulated by alterations in nuclear tension driven by actomyosin contractility.

**Figure 8:**
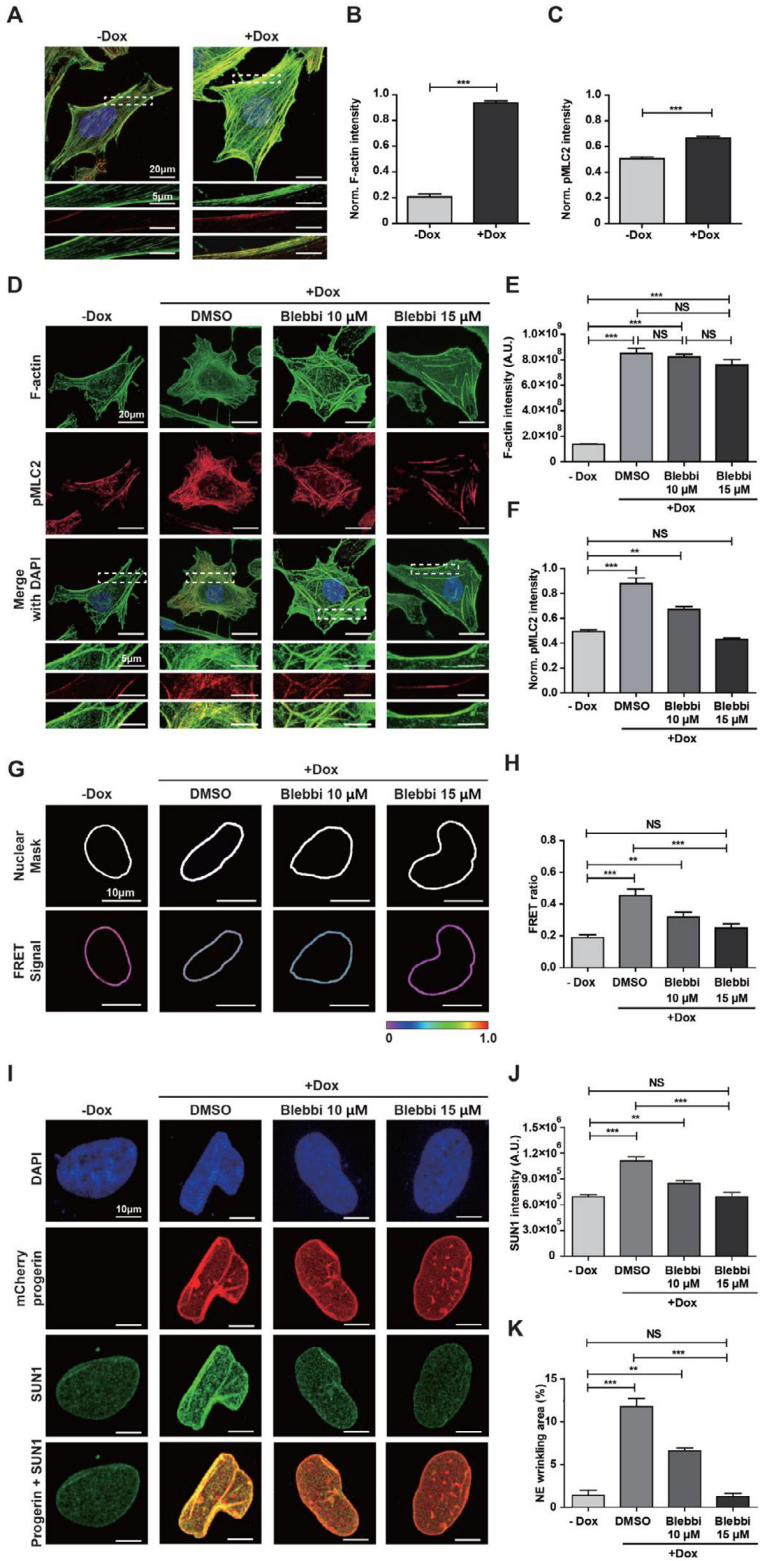
Actomyosin contractility‐dependent nuclear deformation in progerin‐expressing cells. A–C) Differential actomyosin contractility in response to doxycycline‐induced progerin expression. Doxycycline‐inducible progerin‐expressing HeLa cells were immunostained for F‐actin (green), phospho‐myosin light chain 2 (pMLC2, red), and nucleus (DAPI, blue) before (−Dox) and after (+Dox) doxycycline treatment (A). (Insets) The details of pMLC2 staining along the actin stress fibers. Doxycycline‐induced progerin expression significantly increased the F‐actin (B) and pMLC2 (C) contents, which were normalized to cell area and actin stress fibers, respectively. In panels B and C, >60 cells were analyzed for each condition; error bars indicate the S.E.M.; unpaired *t*‐test was applied (***: *p* < 0.001). D–F) Differential formation of F‐actin and pMLC2 in doxycycline‐controlled progerin‐expressing Tet‐On HeLa cells in response to pharmaceutical inhibition of myosin‐dependent cytoskeletal tension. Cells were immunostained for nuclei (DAPI, blue), F‐actin (green), and pMLC2 (red) before (−Dox) and after (+Dox) doxycycline treatment, where differential concentrations of myosin‐II inhibiting blebbistatin were added (D). Compared to −Dox control cells, doxycycline‐induced progerin‐expressing cells showed significantly enhanced F‐actin, which remained unchanged in response to specific disruption of myosin activity (E). Significantly increased pMLC2 content due to doxycycline‐induced progerin expression was reversed by increasing the concentration of blebbistatin (F). Treating doxycycline‐induced progerin‐expressing cells with 15 µm blebbistatin fully restored their pMLC2 content to the level of doxycycline‐untreated progerin nonexpressing cells (F). In panels E and F, >150 cells were analyzed per condition; error bars indicate the S.E.M.; one‐way ANOVA using Tukey's test was applied (***: *p* < 0.001, **: *p* < 0.05, NS: not significant). G,H) Actomyosin contractility‐dependent differential changes of nuclear tension. Representative nesprin tension sensor‐based FRET signals along the nuclear membrane of doxycycline‐inducible progerin‐expressing cells were captured before (−Dox) and after (+Dox) doxycycline treatment in the presence of DMSO and 10 or 15 µm blebbistatin (G). Compared to doxycycline‐untreated control, doxycycline‐induced progerin expression significantly enhanced the FRET ratio, which was gradually diminished by increasing the concentration of blebbistatin and fully restored to the level of doxycycline‐untreated control condition by 15 µm blebbistatin treatment (H). I–K) Tight regulation of SUN1 expression and NE wrinkling in response to changes in actomyosin contractility. Tet‐On HeLa cells expressing mCherry‐tagged Δ50 LMNA (progerin) were treated with DMSO and 10 or 15 µm blebbistatin in the absence (−Dox) and presence (+Dox) of doxycycline before immunostaining for nucleus (DAPI, blue) and SUN1 (green) (I). Compared to the doxycycline‐untreated control, SUN1 expression and NE wrinkling were significantly increased in doxycycline‐treated progerin‐expressing cells, which was gradually diminished by increasing the concentration of blebbistatin and fully restored to the level of the control condition by 15 µm blebbistatin treatment (J,K). In panels H, J, and K, >20 cells were analyzed per condition; error bars indicate the S.E.M.; one‐way ANOVA using Tukey's test was applied for comparison between groups (***: *p* < 0.001, **: *p* < 0.05, NS: not significant).

To investigate whether doxycycline‐controlled progerin expression could alter cytoskeletal tension, we examined F‐actin content and myosin activity using quantitative immunofluorescence microscopy (**Figure** **8**A–C). As predicted, progerin‐expressing cells exhibited increased F‐actin organization (Figure 8A,B). Furthermore, we observed a significant increase in myosin II content relative to F‐actin, assessed by measuring the intensity of phospho‐myosin light chain 2 (pMLC2) normalized to individual F‐actin fibers (Figure 8C). These findings confirm that myosin‐dependent cytoskeletal tension is elevated in progerin‐expressing cells.

Next, to determine whether actomyosin contractility regulates nuclear tension‐mediated NE wrinkling, we treated progerin‐expressing cells with the myosin II inhibitor blebbistatin at varying concentrations (Figure 8D). Consistent with previous results (Figure 8A–C), doxycycline‐induced progerin expression significantly increased F‐actin and pMLC2 intensities in dimethyl sulfoxide (DMSO)‐treated control cells (Figure 8E,F). However, pMLC2 content gradually decreased with increasing drug concentration, and 15 µm blebbistatin treatment fully restored pMLC2 content to the level observed in doxycycline‐untreated progerin nonexpressing cells (Figure 8F), while F‐actin content remained unchanged (Figure 8E).

We assessed actomyosin contractility‐dependent nuclear tension using the nesprin tension sensor in doxycycline‐induced progerin‐expressing cells treated with varying concentrations of blebbistatin (Figure 8G). Surprisingly, we found that a gradual reduction in myosin activity enhanced nuclear tension, as indicated by the FRET ratio (Figure 8H). Treatment of doxycycline‐induced progerin‐expressing cells with 15 µm blebbistatin reduced pMLC2 expression to levels observed in doxycycline‐untreated progerin nonexpressing cells (Figure 8F) without disrupting the F‐actin content (Figure 8E). Under these conditions, the FRET ratio was fully restored (Figure 8H).

We further confirmed that enhanced nuclear tension (i.e., decreased FRET ratio) in response to pharmaceutical inhibition of pMLC2 expression suppressed *SUN1* expression in progerin‐expressing cells (Figure 8I,J). Consequently, the level of NE wrinkling in 15 µm blebbistatin‐treated progerin‐expressing cells was fully restored to that observed in doxycycline‐untreated cells (Figure 8K). These data suggest that inhibition of myosin‐dependent cytoskeletal tension reverses nuclear deformation by downregulating *SUN1* expression and restoring NE tension.

Taken together, our results demonstrate that actomyosin contractility regulates nuclear deformation through alterations in nuclear tension in progerin‐expressing cells.

### 2.9 Inhibition of SUN1 Recovers Progerin‐Induced Nuclear Wrinkling in HGPS Cells

Upregulated SUN1 expression in progerin‐expressing cells resulted in the remodeling of molecular connections with LMNA (Figure 7), and progerin‐induced nuclear deformation was regulated by pMLC2‐dependent nuclear tension (Figure 8). Together with a previous study showing that SUN1 depletion reduces actomyosin activity without disrupting the expression of nesprin 2 in vascular smooth muscle cells,^[^ ^17a^ ^]^ we hypothesized that the level of SUN1 expression in progerin‐expressing cells could determine myosin‐dependent cytoskeletal tension, ultimately regulating nuclear tension‐dependent nuclear deformation.

**Figure 9:**
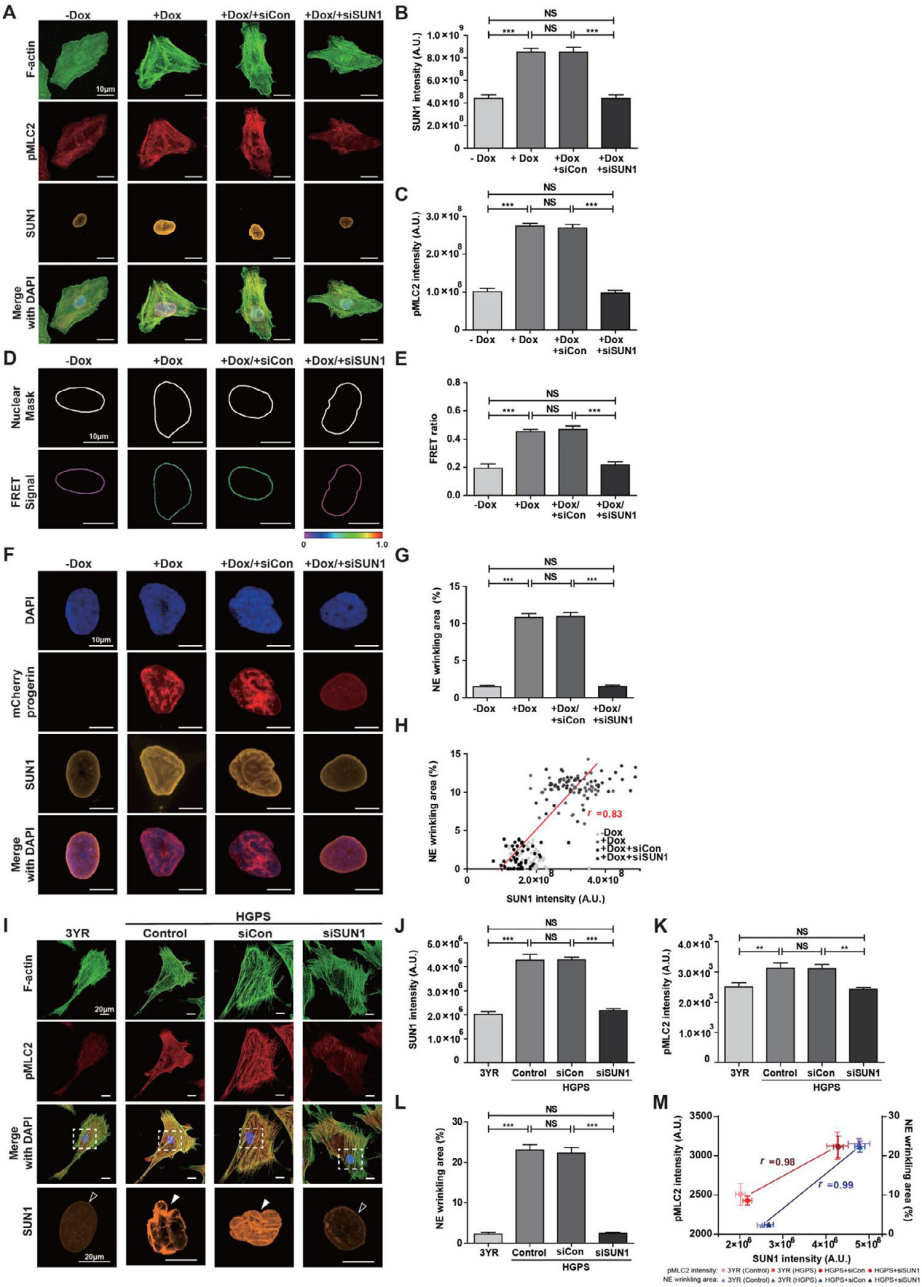
SUN1‐mediated nuclear tension regulates progerin‐induced nuclear deformation. A–C) SUN1‐mediated modulation of actomyosin activity in progerin‐expressing cells. Tet‐On HeLa cells expressing progerin were immunostained for F‐actin (green), pMLC2 (red), SUN1 (orange), and nucleus (DAPI, blue) in the doxycycline‐untreated control condition (−Dox) and doxycycline‐treated conditions (+Dox) with siControl (+Dox/+siCon) or siSUN1 (+Dox/+siSUN1)‐mediated knockdown (A). Doxycycline‐induced progerin expression significantly increased the expression levels of SUN1 and pMLC2, which were maintained in siControl‐transfected cells but restored to levels similar to those observed in doxycycline‐untreated progerin nonexpressing cells after transfection with siSUN1 (B,C). In panels B and C, >150 cells were analyzed per condition; error bars indicate the S.E.M.; one‐way ANOVA using Tukey's test was applied (***: *p* < 0.001, NS: not significant). D–E) SUN1 expression‐dependent NE tension. Nesprin tension sensor‐based FRET signals along the nuclear membrane of doxycycline‐inducible progerin‐expressing cells transfected with siControl (+siCon) or siRNA targeting SUN1 (+siSUN1) were captured before (−Dox) and after (+Dox) doxycycline treatment (D). Doxycycline‐induced enhanced NE tension was maintained in siControl‐transfected cells but restored to the level of the doxycycline‐untreated control condition in siSUN1‐transfected cells (E). In panel E, >20 cells were analyzed per condition; error bars indicate the S.E.M.; one‐way ANOVA using Tukey's test was applied (***: *p* < 0.001, NS: not significant). F–H) Quantification of SUN1‐mediated NE wrinkling. mCherry‐tagged progerin‐expressing Tet‐On HeLa cells transfected with siControl (+siCon) or siRNA targeting SUN1 (+siSUN1) were immunostained for lamin B1 (green), SUN1 (yellow), and nuclear DNA (DAPI, blue) in the absence (−Dox) or presence (+Dox) of doxycycline (F). Doxycycline‐induced progerin expression significantly increased the NE wrinkling, which was maintained in siControl‐transfected cells but reduced to the level of the doxycycline‐untreated control condition in siSUN1‐transfected cells (G). The Pearson product‐moment correlation assessment applied to the merged dataset, including all conditions, showed a highly correlative relationship between SUN1 expression and NE wrinkling (*r* = 0.83) (H). In panels G and H, >50 cells were analyzed per condition; error bars indicate the S.E.M.; and one‐way ANOVA using Tukey's test was applied for comparison between groups (***: *p* < 0.001, NS: not significant). I–M) SUN1‐mediated restoration of the nuclear morphology of HGPS fibroblasts. Human dermal fibroblasts obtained from a three‐year‐old healthy control (denoted by 3 YR) and an HGPS patient were immunostained for F‐actin (green), pMLC2 (red), SUN1 (orange), and nuclei (DAPI, blue), where HGPS fibroblasts were transfected with siControl (HGPS/+siCon) or siSUN1 (HGPS/+siSUN1) (I). Full and empty arrowheads indicate the smooth and wrinkled nuclear surface, respectively. Compared to control fibroblasts, HGPS cells displayed a significantly enhanced expression of SUN1 and pMLC2, which was maintained in siControl‐transfected cells, but transfection with siSUN1 restored SUN1 and pMLC2 expression to levels similar to those observed in control cells (J,K). Nuclear wrinkles specifically featured in HGPS fibroblasts and siControl‐transfected HGPS fibroblasts were recovered in siSUN1‐transfected cells to levels comparable to those in healthy controls (L). Pearson correlation analysis applied to the merged dataset incorporating all experimental conditions showed a strong correlation between SUN1 expression and pMLC2 expression (red, *r* = 0.98), SUN1 expression, and NE wrinkling (blue, *r* = 0.99) (M). In panels J and K, >50 cells were analyzed per condition; in panel L, >20 cells were analyzed per condition; error bars indicate the S.E.M.; one‐way ANOVA using Tukey's test was applied for comparison between groups (***: *p* < 0.001, **: *p* < 0.05, NS: not significant).

To directly assess the causality between *SUN1* expression and myosin activity, we inhibited *SUN1* expression by transfecting progerin‐expressing Tet‐On HeLa cells with small interfering RNA (siRNA) and compared them to control siRNA‐transfected cells (**Figure** **9**A). Consistent with previous data (Figures 7K and 8C), doxycycline‐induced progerin‐expressing cells transfected with control siRNA showed significantly increased levels of *SUN1* and pMLC2, whereas siRNA‐mediated *SUN1* depletion reduced these levels comparable to those observed in doxycycline‐untreated, progerin‐negative cells (Figure 9B,C). Confirming that the changes in pMLC2 expression in Tet‐On HeLa cells in response to doxycycline‐induced progerin expression and/or siRNA transfection were largely proportional to changes in *SUN1* expression (Figure 9A–C), a strong correlation between *SUN1* and pMLC2 expression was detected, regardless of specific conditions (Figure S9, Supporting Information). These results indicate that increased pMLC2 expression in progerin‐expressing cells is induced by *SUN1* upregulation.

As reduced nuclear tension (i.e., increased FRET ratio) was restored by inhibiting pMLC2 expression (Figure 8H), we assessed whether transfection with SUN1‐siRNA could also restore this reduced nuclear tension (Figure 9D). In progerin‐expressing cells transfected with SUN1‐siRNA (+Dox/+siSUN1), the increased FRET ratio induced by doxycycline‐mediated progerin expression (+Dox or +Dox/+siCon) was reduced to the level observed in doxycycline‐untreated cells (−Dox) (Figure 9E). This confirmed that the reduced nuclear tension in progerin‐expressing cells was mediated by *SUN1* upregulation.

Measurement of NE wrinkling indicated that nuclear deformation caused by progerin‐induced reduction in nuclear tension was reversed by transfection with SUN1‐siRNA (Figure 9F,G), indicating a strong correlation between *SUN1* content and the level of NE wrinkling under all conditions (Figure 9H). Combined with previous data showing that progerin‐induced NE wrinkling is mediated by a SUN1‐dependent reduction in nuclear tension, accompanied by upregulated myosin‐associated cytoskeletal tension (Figures 7 and 8), and that gradual inhibition of pMLC2 expression reverses the upregulation of *SUN1* and NE wrinkling (Figure 8J,K), these findings suggest that *SUN1* upregulation is responsible for nuclear deformation in progerin‐expressing cells.

To extend our findings to human patients suffering from an accelerated/premature aging disorder, we utilized dermal fibroblasts derived from a 3‐year‐old patient with HGPS and compared them to control cells from a healthy individual of the same age. Transfection of HGPS fibroblasts with SUN1–siRNA significantly reduced the expression of both SUN1 and pMLC2, without disrupting F‐actin organization, which remained unchanged over 5 days (Figure S10, Supporting Information). By systematically comparing fibroblasts from healthy individuals (denoted as 3 YR), we found that both HGPS fibroblasts and siControl‐transfected HGPS fibroblasts exhibited significantly increased levels of SUN1 and pMLC2, which was reversed by transfection with siSUN1 (Figure 9I–K). Accordingly, the level of NE wrinkling, which is specifically featured in HGPS fibroblasts and siControl‐transfected HGPS fibroblasts, was restored to healthy control levels by siRNA‐induced depletion of SUN1 (Figure 9I,L). In patients with HGPS, a stronger correlation was observed between SUN1 and pMLC2 intensities, as well as nuclear envelope wrinkling. Notably, SUN1 knockdown using siRNA reduced these levels to those observed in healthy controls (Figure 9M).

**Figure 10:**
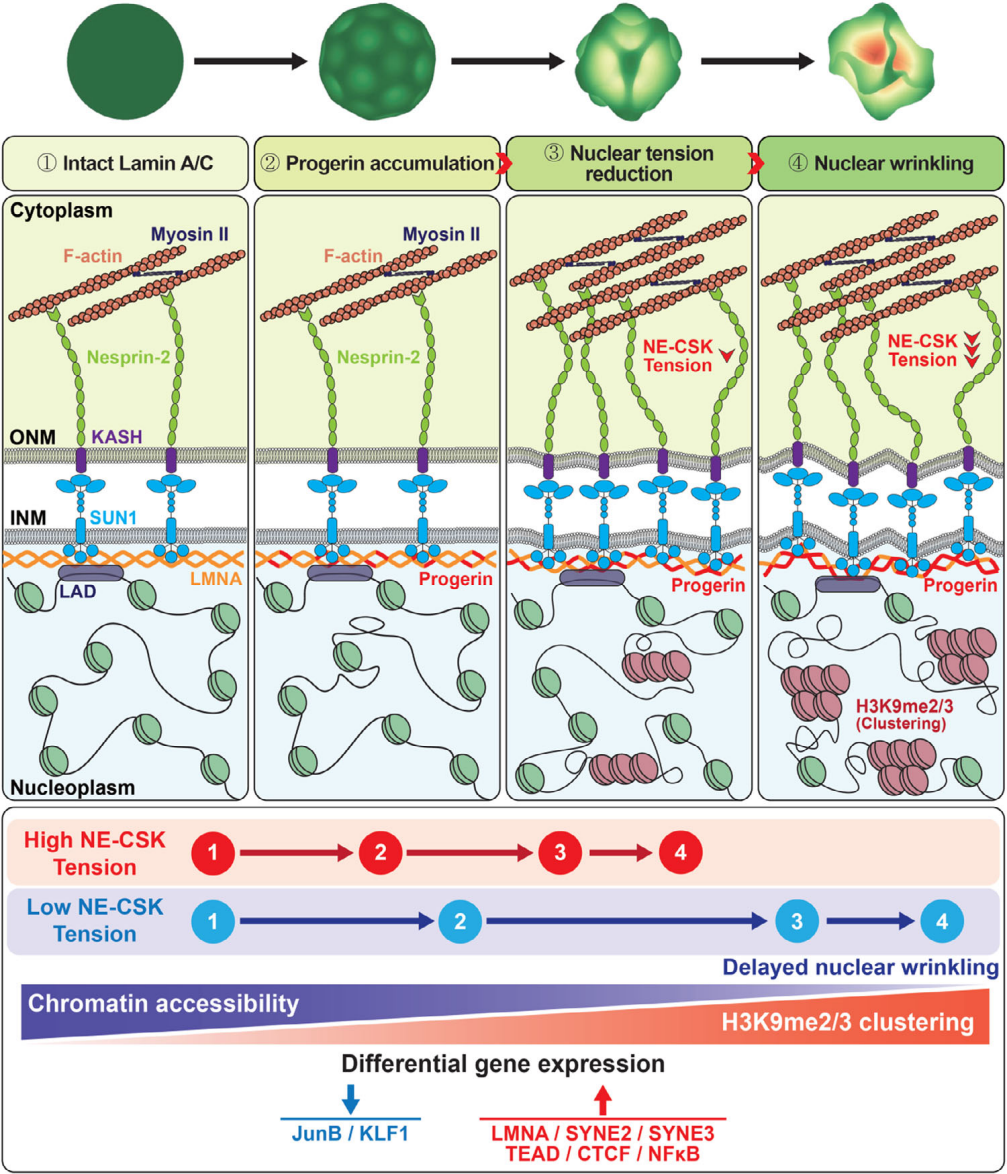
Schematic summary depicting the functional relationship between SUN1‐mediated nuclear tension and NE wrinkling in response to progerin expression. Progerin expression accumulates LINC complex proteins SUN1 and Nesprin 2, reorganizing the actin‐binding Nesprin‐associated LINC complex at the nuclear envelope, and determining the biophysical interactions of the nuclear–cytoskeletal connection. Although the molecular linkages connecting SUN1, Nesprin 2, and F‐actin remain unchanged in response to progerin expression, nuclear tension along the SUN1–Nesprin 2–F‐actin connection is reduced by increased pMLC2. In summary, progerin‐induced morphological defects forming the surface wrinkling along the nuclear lamina are determined by the accumulation of LINC complexes proteins at the nuclear envelope and reduced nuclear tension accompanied by pMLC2 via the SUN1–Nesprin 2 bridge, regulating the expression of various genes within the nucleus. Ultimately, progerin‐induced nuclear wrinkling features increased chromatin dynamics in the heterochromatin‐rich nuclear periphery, resulting in the misregulation of mechanotransduction signal pathways in the HGPS model. Doxycycline‐induced progerin expression exhibits mechanical sensitivity to variations in substrate stiffness. Approximately 10%, 25%, and 28% of delays in onsets of progerin expression, reduction of nuclear tension, and nuclear wrinkling, respectively, on the soft substrate identifies the intracellular cytoskeletal force exerted on the nucleus as the origin of progerin‐induced nuclear wrinkling.

Together with previous results showing that doxycycline‐induced progerin expression results in *SUN1* accumulation in the nuclear lamina, where nuclear tension along the *SUN1*–nesprin 2–F‐actin connections is diminished by increased pMLC2 in response to progerin expression, these results reconfirm that defective nuclear morphology in HGPS is induced by reduced nuclear tension, accompanied by *SUN1* upregulation‐mediated pMLC2 expression rather than altered F‐actin connectivity, which coincides with chromosomal remodeling via modification of heterochromatin accessibility (**Figure** **10**).

## 3 Discussion

Increased cytoskeletal tension, determined by its connection to the nuclear envelope, is crucial in regulating the mechanical forces transmitted to the nucleus. However, the causal relationship between progerin‐induced changes in cytoskeletal dynamics and nuclear deformation in progerin‐expressing cells remains unclear. In this study, we aimed to address this gap by investigating the molecular mechanisms underlying nuclear morphological changes in response to progerin expression and their contribution to chromatin reorganization and aberrant gene expression patterns observed in HGPS. In this study, we employed a doxycycline‐inducible Tet‐On system to precisely control progerin expression in a human HGPS cell model, enabling real‐time monitoring through mCherry‐tagged proteins. This system facilitates the spatiotemporal monitoring of nuclear deformation in response to mechanosensitive progerin expression while overcoming the limitation of primary cell availability. This approach enabled us to investigate the molecular mechanisms underlying progerin‐induced nuclear deformation and its impact on temporal chromatin remodeling and gene expression profiles. Moreover, this system served as an experimental platform to explore the relationship between progerin‐induced nuclear deformation and mechanosensing of substrate stiffness.

In combination with previous reports showing that adhesion‐dependent cells typically display enhanced cytoskeletal tension on a rigid matrix compared to that on a compliant matrix to maintain mechanical integrity,^[^ ^55^ ^]^ our findings of enhanced myosin activity in progerin‐expressing cells indicate that the signaling pathways determining the progerin‐induced nuclear deformation are highly mechanosensitive (Figure 1). Previous results showed that dermal fibroblasts from a patient with HGPS exhibited abnormal nuclear morphology and even formed nuclear rupture on stiff substrates ranging from 10 to 20 kPa and 80 kPa, whereas such abnormalities were less frequently observed in soft substrates ≈3 kPa.^[^ ^56^ ^]^ Because mechanosensitive nuclear deformation is also time‐dependent (Figure 3), These results suggests that the differential onset of nuclear abnormalities correlates with an increase in cytoskeletal tension, featuring enhanced cell spreading and elongation that typically amplify cytoskeletal forces transmitted to the nucleus. Since progerin expression is regulated by cytoskeletal forces, nuclear responses to differential expression of progerin vary depending on the mechanical properties of the extracellular environment. Thus, exposure to substrates of varying substrate stiffness could modulate cytoskeletal tension and mechanotransduction, ultimately shaping the cellular adaptations associated with progerin‐induced phenotypes.^[^ ^57^ ^]^ As varying matrix rigidity induces distinct differentiation of MSCs, e.g., neurogenesis on soft substrates ranging from 0.1 to 1 kPa, and osteogenesis on stiff substrates ranging from 25 to 40 kPa, respectively,^[^ ^58^ ^]^ 1.37 kPa condition applied in our study closely mimics physical settings of soft tissues, whereas 34 kPa condition represents a microenvironment of stiffer tissues (Figure 1). This enables our system to reliably predict the phenotypic development associated with nuclear deformation observed in HGPS patients.

We observed that progerin expression induced a critical reduction in nuclear tension after 24 h, followed by NE wrinkling within 1 h on stiff substrates, whereas these time intervals doubled on soft substrates (Figures 1 and 2). These results indicate that the synergistic increase in cytoskeletal tension can be attributed to the combination of progerin expression and plating of cells on the rigid matrix, which accelerates the individual steps of progerin‐induced reduction in nuclear tension and nuclear tension‐dependent NE wrinkling (Figures 1 and 2). The computational model system further demonstrated that as the mechanical resistance of the nucleus to external pressure increased (i.e., on a stiff substrate), the nuclear volume and NE tension decreased, leading to accelerated nuclear wrinkling compared to the nucleus on the soft substrate (Figure 3).

Meanwhile, RNA sequencing comparing the number of DEGs following progerin expression on substrates of varying elastic moduli showed that the largest shift in gene expression levels (both upregulation and downregulation) occurred at 24 h after doxycycline treatment on stiff substrates and at 30 h on soft substrates (Figure 4A,B), following a significant reduction in NE tension (Figures 1 and 2). These results indicate that progerin‐induced nuclear wrinkling is not only mechanosensitive to changes in extracellular mechanical settings, mainly mediated by actomyosin contractility and nucleus–cytoskeletal connections,^[^ ^16^ ^]^ but also induces distinct pathogenetic gene regulation. The upregulation of *LMNA*, *SYNE2*, and *SYNE3* supports this notion that mechanosensitive progerin expression mediates essential signaling pathways and functional gene regulation (Figure 4). Moreover, consistent with enhanced expression of H3K9me2/3 on rigid substrates,^[^ ^46^ ^]^ progerin‐induced epigenetic modification is highly mechanosensitive (Figure 5) and leads to enhanced chromatin dynamics in fibroblasts from patients with HGPS (Figure 6A–C).

A comparative analysis with publicly available RNA sequencing datasets from primary fibroblasts of healthy individuals and patients with HGPS (GSE141950 and GSE118633) revealed distinct gene expression profiles in HGPS fibroblasts (Figure S6, Supporting Information). Pathway enrichment analyses further demonstrated consistent alterations in cellular components, biological processes, and molecular functions across models. Notably, the GSE118633 dataset corroborated pathway‐level changes, reinforcing the validity of our findings. These results not only support the robustness of our Tet‐On‐inducible progerin‐expressing HeLa cell model in recapitulating key molecular features of HGPS but also suggest that progerin expression differentially regulates gene expression in a substrate stiffness‐dependent manner by modulating multiple signaling pathways (Figure 4D–H; Figure S6, Supporting Information). In addition, ATAC‐seq revealed that the progerin‐induced alteration of transcription factors is also responsive to substrate stiffness (Figure 6D–R), further suggesting that progerin‐induced nuclear wrinkling amplifies chromatin dynamics by promoting heterochromatin clustering to regulate chromatin accessibility.

Intact LMNA‐producing normal cells typically transmit cytoskeletal tension to the nuclear membrane via the stable expression of LINC complex‐associated molecular components.^[^ ^16^ ^]^ Therefore, a strong correlation between cytoskeletal tension and the force applied to the nucleus has been detected.^[^ ^59^ ^]^ However, we noted a mismatch between the reduced nuclear tension and enhanced cytoskeletal tension in doxycycline‐induced progerin‐expressing cells (Figure 2 vs Figure 8). This could be due to the formation of the *LMNA* mutant‐progerin, which could disrupt the *LMNA*‐mediated molecular connection. Consequently, the cytoskeletal forces may not be properly transmitted into the nucleus, and vice versa. The mechanical imbalance between outer nuclear cytoskeletal tension and LINC‐mediated nuclear tension in progerin‐expressing cells further highlights the critical role of *LMNA*‐dependent molecular connections between the NE and cytoskeleton. Notably, we showed that doxycycline‐induced progerin expression upregulated LINC complex‐associated genes (Figure 7).

Previous studies have shown that progerin predominantly associates with *SUN1*, but not with *SUN2*, in LMNA^−/−^ mouse embryonic fibroblasts,^[^ ^28^ ^]^ and that the stable expression of progerin proportionally increases the level of *SUN1*, but not *SUN2*, in progerin‐expressing NIH3T3 fibroblasts.^[^ ^26b^ ^]^ In an in situ PLA assay, we observed that individual interactions between LMNA and SUN1 were increased in response to progerin expression, while the molecular interaction between *SUN1* and its binding partner nesprin 2 remained unchanged (Figure 7O–T). Combining these results, we propose a new model that fills the missing link between myosin activity‐dependent cytoskeletal tension and *SUN1*‐mediated nuclear tension (Figure 8). Our findings suggest that progerin‐induced nuclear deformation is mediated by reduced nuclear tension, accompanied by *SUN1* upregulation‐dependent myosin tension, rather than by F‐actin connectivity through *SUN1*–nesprin 2 bridging. Validation in both SUN1‐depleted Tet‐On HeLa cells and HGPS patient‐derived fibroblasts established that our HeLa cell models are consistent with those from primary HGPS fibroblasts. This concordance reinforces that SUN1‐dependent nuclear tension plays a critical role in regulating progerin‐induced nuclear deformation, highlighting the mechanistic relevance of our cell model to recapitulate key aspects of pathological features of HGPS (Figure 9).

Taken together, our results suggest that i) progerin expression, reduced NE tension, and nuclear wrinkling are mechanosensitive to changes in substrate stiffness, ii) progerin expression disrupts the *LMNA*‐mediated force balance between cytoskeletal force and nuclear tension, iii) the SUN1–*LMNA* interaction mediates force transmission from the cytoskeleton to the nuclear interior, and iv) reduced nuclear tension enhances chromatin dynamics, thereby regulating progerin‐induced mechanosensitive signaling pathways. The key findings regarding the SUN1‐dependent nuclear tension and chromatin remodeling have important translational implications, as they suggest that manipulation of nuclear tension could provide a novel therapeutic strategy to mitigate the effects of progerin‐induced nuclear deformation. These results could pave the way for developing targeted interventions that modulate nuclear mechanics to treat progeria as well as diseases associated with defects in nuclear architecture.

## 4 Experimental Section

### Cell Culture and Drug Treatment

HeLa cells (purchased from Korean Cell Line Bank, Seoul, Republic of Korea) were cultured in T25 rectangular canted neck cell culture flasks (Falcon, 353108) containing 2 mL of Dulbecco's Modified Eagle's Medium (DMEM, Corning, 10‐013‐CV) supplemented with 10% fetal bovine serum (FBS, Merck, TMS‐031‐BKR) and 1% penicillin–streptomycin (Thermo, 15140122) at 37 °C with 5% CO_2_ in a humidified incubator. Human dermal fibroblasts obtained from a 3‐year‐old normal healthy individual (GM05565) and a 3‐year‐old HGPS patient (AG06917) were purchased from Coriell Cell Repositories (Camden, NJ). Fibroblast cells were cultured in DMEM supplemented with 15% FBS and 1% penicillin–streptomycin at 37 °C with 5% CO_2_ in a humidified incubator. Culture media used in this work were refreshed every 2–3 days. To inhibit myosin II activity, HeLa cells were treated with 10 and 15 µm blebbistatin (Sigma‐Aldrich, B0560) for 1 h. Control cells, without blebbistatin, were treated with DMSO (Merck, 317275) and incubated under identical conditions. After DMSO or blebbistatin treatment, fresh culture medium was added prior to immunostaining and imaging.

### Plasmids and Subcloning

To generate stable cell lines with doxycycline‐inducible expression of wild type LMNA or Δ50 LMNA tagged with/without mCherry, PCR‐amplified DNA sequences for mCherry‐LMNA (Addgene, #55068), mCherry‐Δ50 LMNA (modified from Addgene, #17653), and Δ50 LMNA (modified from Addgene, #17653) were inserted into PiggyBac XLone‐GFP plasmid (Addgene, #96930) after digesting with KpnI, SpeI to replace the GFP sequence.^[^ ^36^ ^]^ To deliver genetic cargo into the genome, blasticidin (Thermo Fisher, R21001)‐resistant gene‐containing PiggyBac transposon was used.^[^ ^60^ ^]^ Telomeric repeat‐binding factor 2 (TRF2)‐GFP plasmid used for analyzing chromatin dynamics was modified. PCR‐amplified DNA sequences for TRF2 were obtained using TRF2‐IRES‐eGFP (Addgene, #19798), and PCR‐amplified DNA sequences for pcDNA3‐EGFP (Addgene, #13031). The TRF2 and pcDNA3‐EGFP were ligated using the NEBuilder HiFi DNA assembly master mix (NEB, E2621L) to construct the EGFP‐TRF2 plasmid.

### Tet‐On System for Doxycycline‐Inducible Gene Expression

HeLa cells were seeded in T25 rectangular canted neck cell culture flasks to reach 70–80% confluency after culturing for 24 h in DMEM supplemented with 10% FBS and 1% penicillin–streptomycin at 37 °C and 5% CO_2_ in a humidified incubator. The cells were then transfected with the generated plasmids (1 µg µL^−1^; mCherry‐LMNA, mCherry‐Δ50 LMNA, or Δ50 LMNA) and the PiggyBac transposase (0.5 µg µL^−1^; System Biosciences, PB210PA‐1) using Lipofectamine 3000 (Thermo Fisher, L3000015) following the supplier's instructions. After 72 h of incubation, the cells were replated onto 96‐well cell culture plates (SPL, 30096) and selected with 5 µg mL^−1^ blasticidin for 7 d to generate stable cell lines expressing the respective target genes. To induce gene expression, HeLa cells transfected with mCherry‐lamin A‐, mCherry‐Δ50 lamin A‐, or Δ50 lamin A were treated with 2 µg mL^−1^ doxycycline (Merck, D9891).

### Immunofluorescence and Time‐Lapse Live Cell Monitoring

After growing for 24 h in a glass bottom dish (SPL, 101350) coated with 0.2 mg mL^−1^ type‐I rat tail collagen (Corning, 354236) diluted in 0.2 N acetic acid, the cells were fixed with 4% paraformaldehyde (Biosesang, PC2031‐100‐00) for 10 min at 4 °C, permeabilized with 0.1% Triton X‐100 (Merck, T8787) for 10 min, and then blocked with phosphate‐buffered saline (PBS, Corning, 21‐031‐CV) supplemented with 10% FBS for 30 min at room temperature (RT). Fixed cells were incubated with primary antibodies for 1 h at RT. After washing with PBS three times, secondary antibodies with 4′,6‐diamidino‐2‐phenylindole dihydrochloride (DAPI, Sigma‐Aldrich, D1306) and Alexa Fluor 488 phalloidin (Thermo, A12379) were added. Primary antibodies used in this study are as follows: anti‐Progerin (1:200, Abcam, ab66587), anti‐Lamin B1 (1:500, Abcam, ab16048), anti‐SUN1 (1:200, Millipore, ABT273), anti‐Nesprin 2 (1:100, Sigma‐Aldrich, MABC86), anti‐phospho‐myosin light chain 2 (Ser19) (1:200, CST, 3675), and di/tri‐methyl‐histone H3 (Lys9) (1:100, CST, 5327). Secondary antibodies used are as follows: Goat anti‐Mouse IgG Heavy and Light Chain Antibody DyLight 488 Conjugated (Bethyl, A90‐116D2), Sheep anti‐Rabbit IgG Heavy and Light Chain Antibody DyLight 488 Conjugated (Bethyl, A120‐100D2), Goat anti‐Mouse IgG‐Heavy and Light chain Antibody DyLight 594 Conjugated (Bethyl, A90‐116D4), Sheep anti‐Rabbit IgG‐Heavy and Light chain Antibody DyLight 650 Conjugated (Bethyl, A120‐100D5). All samples were imaged using confocal laser microscopy (A1R, Nikon) through 20x plan lens with a z‐stack of 0.4 µm or 60× oil lens with a z‐tack of 0.2 µm or using fluorescence microscopy (Ti2, Nikon). For time‐lapse live cell monitoring, HeLa cells expressing mCherry‐Δ50 lamin A were seeded onto a glass bottom dish and imaged through a 20× plan lens using a confocal microscope equipped with a stage‐top incubator (Okolab, Italy). Live cell images were captured every 20 min for 36 h to monitor progerin expression and NE wrinkling, and every 30 min for 16 h to collect z‐stacked NE wrinkling images. Z‐stacked time‐lapse confocal images were reconstructed and 3D images were rendered using NIS elements software (Nikon).

### Nuclear Morphometry and Protein Content Measurement

High‐throughput cell phenotyping analysis was employed to assess cell and nuclear size, nuclear aspect ratio, nuclear circularity, and protein expression at a single‐cell level.^[^ ^61^ ^]^ Briefly, immunostained cells were autofocused through the DAPI channel, and multiple images were automatically captured on a scale of 7 × 7 in two or three channels with DAPI, fluorescein‐5‐isothiocyanate (FITC), and tetramethylrhodamine through a 20× plan lens using a fluorescence microscope. Image analysis was conducted using a customized program coded in MATLAB (MathWorks Laboratory). In the acquired images, a threshold value was applied to each FITC and DAPI channel image to separate the cell and nuclear regions, prior to calculation of the fluorescence intensity. To determine the nuclear deformation, the fractional occupancy of nuclear wrinkles shown as the bright regions in the nucleus marked by expression of mCherry‐Δ50 lamin A or immunostaining of Lamin B1 was quantified. Specifically, the degree of NE wrinkling was calculated using the following equation

(1) $$\mathrm{NE}\;\mathrm{wrinkling}\;\left(\%\right)=\frac{\left(\mathrm{Nuclear}\;\mathrm{wrinkling}\;\mathrm{area}\right)}{\left(\mathrm{Nuclear}\;\mathrm{spreading}\;\mathrm{area}\right)}\times 100$$

where the nuclear wrinkling area and the nuclear spreading area indicate the nuclear region showing a higher intensity value than the average intensity value of the entire nucleus and the area of the fluorescence‐marked entire nucleus, respectively.

### Preparation of Polyacrylamide Hydrogel Substrates

The surface of glass bottom dishes was pretreated with (3‐aminopropyl) trimethoxysilane (Sigma‐Aldrich, 281778) for 5 min and then with 0.5% glutaraldehyde (Sigma‐Aldrich, G6257) for 30 min. After washing with deionized water, acrylamide/bis‐acrylamide solution containing ammonium persulfate (Sigma‐Aldrich, A3678) and tetramethylenediamine (Invitrogen, 15524‐010) was added onto the surface‐modified glass bottom dish. Dichlorodimethylsilane (Sigma‐Aldrich, 40140) precoated cover slips were immediately placed onto the droplets to form a flat PAG. PAGs were activated with Sulfo‐SANPAH (Thermo Fisher, 22589), followed by coating with type‐I rat tail collagen overnight at 4 °C. To modulate the stiffness of the PAG, the concentration ratios of acrylamide and bis‐acrylamide were varied as follows: 5% acrylamide + 0.06% bis‐acrylamide (*E* ≈ 1.37 kPa) and 10% acrylamide + 0.3% bis‐acrylamide (*E* ≈ 34 kPa). The elastic moduli of the gels were adapted from previous studies.^[^ ^62^ ^]^

### Construction of Nuclear Tension Sensor

To measure nuclear tension, a previously developed nesprin tension sensor was modified (Addgene, #68127).^[^ ^63^ ^]^ Briefly, the FRET‐based nesprin tension sensor consisted of mTEP1 and venus, which were separated by a 40‐amino acid elastic linker flanked by XhoI and NotI restriction sites. To modify the nesprin tension sensor to accommodate the confocal microscopy wavelength, the mTEP1 and venus were replaced with enhanced green fluorescence protein (EGFP) and DsRed, respectively, using the pQCXI Puro DsRed‐LC3‐GFP plasmid (Addgene, #31182).

### FRET Imaging and Analysis

To determine nuclear tension, cells cultured on glass bottom dishes were transfected with a modified nesprin tension sensor construct. EGFP and DsRed were excited by 488 and 561 nm lasers, respectively. FRET signals of cells expressing the nesprin tension sensor were captured at 488 nm excitation wavelength through a 60× oil lens using the Nikon A1R confocal laser microscope equipped with a stage‐top incubator. Images were acquired on the same day at a fixed gain and laser intensities in each channel, and then analyzed using NIS‐Element software. The FRET ratio was defined as the ratio of energy transferred from the donor to the acceptor depending on the distance between the donor and acceptor proteins, as described by the following equation

(2) $$\mathrm{FRET}\;\mathrm{ratio}=\frac{\left(\mathrm{Intensity}\;\mathrm{of}\;\mathrm{DsRed}-\mathrm{Background}\;\mathrm{of}\;\mathrm{DsRed}\right)}{\left(\mathrm{Intensity}\;\mathrm{of}\;\mathrm{EGFP}-\mathrm{Background}\;\mathrm{of}\;\mathrm{EGFP}\right)}$$

The FRET ratio was color‐coded for better visibility.

### Computational Analysis of Nuclear Deformation

A mechanical model of a soft spherical elastic thin shell was developed to characterize the deformation of nuclear surface. The spherical shell is discretized into many (5120 in this work) identical triangle face elements. Each face element contains 3 vertices and 3 sides, for the entire sphere shell, excluding repeated counted vertices, there exist a total of 2562 independent vertices. According to Euler's formula of polyhedral

(3) V−E+F=2

5120 independent sides are defined, where *V* is the number of vertices, *E* is the number of sides, and *F* is the number of faces.

The deformation and motion of the shell are described by the vertices, and the motion of each vertex follows the over damped Langevin equation

(4) $$\eta \;\frac{dr_{i}}{dt}=-\nabla_{i}U+F_{i}^{r}+P_{i}^{e}$$

where *η* is the damping coefficient, ***r*** *_i_* is the position vector of vertex *i*. *U* is the total shape potential energy of the entire shell, and ∇*_i_* means to derive the gradient in terms of ***r*** *_i_*. ***F*** *_i_* ^r^ is the repulsive force exerting onto vertex *i* by other vertices that are too close to it and ***P*** *_i_* ^e^ is the external pressure exerting onto vertex *i* from the environment.

Using the expression of charged elastic shell,^[^ ^64^ ^]^ the shape potential energy *U* of the sphere shell is written as

(5) $$U=U_{e}+U_{b}+U_{V}$$

with

(6) $$U_{e}=\sum_{i=1}^{E}\frac{K_{e}}{2}{\left(l_{i}-l_{0}\right)}^{2}$$

(7) $$U_{b}=\sum_{i=1}^{E}\frac{K_{b}}{2}{\left|{\hat{n}}_{i,1}-{\hat{n}}_{i,2}\right|}^{2}$$

(8) $$U_{V}=\sum_{i=1}^{F}\frac{K_{V}}{2}{\left(V_{i}-V_{0}\right)}^{2}$$

where *U*_e_ represents the stretching energy, generated from the extension of shortening of all sides, *K*_e_ is the stretching modulus, *l_i_* is the actual length of side *i*, *l*_0_ is the initial side length shared by all sides. *U*_b_ represents the bending energy, generated from the bending of the membrane featured by the dihedral angle between each two adjacent faces, *K*_b_ is the bending modulus, ${\hat{n}}_{i,1}$ and ${\hat{n}}_{i,2}$ are the unit outward normal vectors of the 2 adjacent faces of side *i*. *U*_e_ and *U*_b_ describe the mechanical properties of the membrane itself. *U*_V_ represents the volume energy, generated from the elasticity of the contents of the shell, *K*_V_ is the volume modulus, *V_i_* is volume element *i*’s volume defined by face *i*, and *V*_0_ is the initial volume shared by all elements.

***F*** *_i_* ^r^ represents the repulsive forces between 2 very close vertices, and the traditional 6–12 law is adopted to characterize the molecular level interactions

(9) $$F_{i}^{r}=-K_{r}\sum_{j\varepsilon C_{i}}\left(\frac{1}{{\left|r_{i}-r_{j}\right|}^{6}}-\frac{1}{{\left|r_{i}-r_{j}\right|}^{12}}\right)\left(r_{i}-r_{j}\right)$$

where *K*_r_ is the repulsion coefficient, *C_i_* represents the set of other vertices whose distance from vertex *i* is less than a threshold distance *D*.

The cytoskeleton in connected to the nuclear lamina through the LINC complexes, and the LINC complexes are regarded as a molecular spring. Once the progerin is expressed, the distance between cytoskeleton and the nuclear lamina gets reduced (refer to FRET Imaging and Analysis), leading to a reduced nuclear tension, thus the spring can generate greater compressive pressure between the cytoskeleton and nucleus membrane. Hence, a uniform and constant external pressure term ***P***_i_^e^ is used to model the force exerting onto the shell (nucleus membrane) by the environment (cytoskeleton)

(10) $$P_{i}^{e}=-P_{i}^{e}\nabla_{i}V$$

where *P_i_* ^e^ is the scalar value of ***P*** *_i_* ^e^, and *V* is the total volume of the shell. With increasing substrate stiffness, the pressure also increases, therefore, *P_i_* ^e^ is set higher on stiffer substrate. Specifically, *P_i_* ^e^ *=* 500*P*_0_, 600 *P*_0_, and 750 *P*_0_ are set on soft, medium, and stiff substrates, respectively. All parameters and values used in the model were listed in **Table** **1**.

**Table 1:** Parameters for mechanical model.

Parameter	Numerical value	Refs.
*F*	5120	[71]
*V*	2562	
*E*	7680	
*K*_e_	1000	[64]
*K*_b_	4	
*K*_V_	3	
*K*_r_	1 × 10^−13^	
$P_{i}^{e}$	500 on soft substrate	[72]
	600 on medium substrate	
	750 on stiff substrate	
η	1	[73]
d*t*	0.005	
*D*	0.9	

### RNA Sequencing

Cells cultured on glass and 1.37 kPa PA gel were used to extract total RNA using the RNeasy Mini Kit (QIAGEN, 74104). A library was independently prepared with 1 µg of total RNA for each sample by Illumina TruSeq Stranded mRNA Sample Prep Kit (Illumina, Inc., San Diego, CA, USA, #20020595). Libraries were quantified using KAPA Library Quantification kits for Illumina Sequencing platforms according to the qPCR Quantification Protocol Guide (KAPA BIOSYSTEMS, #KK4854) and qualified using a TapeStation D1000 ScreenTape (Agilent Technologies, # 5067–5582). Indexed libraries were then subjected to Illumina NovaSeq (Illumina, Inc., San Diego, CA, USA), and paired‐end (2 × 100 bp) sequencing was performed by Macrogen Inc. (South Korea). Reference genome sequences and gene annotation data were downloaded from the NCBI Genome Assembly and RefSeq databases, respectively. The aligned data (SAM file format) were sorted and indexed using SAM tools v 1.9. After alignment, the transcripts were assembled and quantified using StringTie v2.1.3b.^[^ ^65^ ^]^ Gene‐ and transcript‐level quantification were calculated as the raw read count, Fragments Per Kilobase of transcript per million mapped reads, and transcript per million mapped reads. The statistical significance of the DEGs was determined using the edgeR most exact, and the fold change and *p*‐value were extracted from the most exact results. All *p*‐values were adjusted using the Benjamini–Hochberg algorithm to control the false discovery rate. Hierarchical clustering of the log‐transformed values for significant genes was performed using these parameters (distance metric = Euclidean distance; linkage method = complete). Gene‐enrichment and functional annotation analysis for significant genes were carried out using gProfiler (Raudvere, Uku, et al. 2019, https://biit.cs.ut.ee/gprofiler/orth) against Gene Ontology (GO) database. Adjusted *p*‐values reported from the gProfiler results were derived using a one‐sided hypergeometric test and corrected using the Benjamini–Hochberg method.

### Assay for Transposase‐Accessible Chromatin Using Sequencing (ATAC Sequencing)

For ATAC sequencing, samples were prepared according to previously established methods. Briefly, cells were detached from the glass and 1.37 kPa polyacrylamide hydrogel; 100 000 cells were prepared. The cells were then lysed in a cold lysis buffer. The nuclei concentration was determined using LUNA‐FL Automated Fluorescence Cell Counter (logos biosystems) and the nuclei morphology was examined using microscopy. Immediately after lysis, the transposition reaction was continued using Tagment DNA TDE1 Enzyme and Buffer Kit (Illumina). Nuclei (50 000 cells) were resuspended in the transposition reaction mixture by incubating for 30 min at 37 °C. Immediately after transposition, cells were purified using the Qiagen MinElute PCR Purification Kit. ATAC‐seq was performed by Macrogen, Inc. (South Korea). The cleaned reads were aligned to the human genome (GRCh38) using Bowtie2.^[^ ^66^ ^]^ The mapped data (in SAM file format) were sorted and indexed using SAM tools (version 1.9).^[^ ^67^ ^]^ After removing the reads aligned to the mitochondrial genome from the indexed BAM file, duplicate reads were removed using MarkDuplicates in Picard (version 0.118). Peaks in the aligned sequence data were identified using a model‐based analysis of ATAC‐seq (MACS2 version 2.1.1.20160309).^[^ ^68^ ^]^ The algorithm empirically models the length of ATAC‐seq fragments from sequence data, considering local genomic biases in the distribution of mapped reads. The following parameters were used: macs2 callpeak‐t ATAC‐seq. bam‐g hs–bdg–nolambda–keep‐dup all–broad. Among the called peaks, those that overlapped with the ENCODE blacklisted regions were excluded. ChIPseeker (version 1.22.1),^[^ ^69^ ^]^ a Bioconductor package in R (version 4.2.2) used to facilitate batch annotation of enriched peaks, was used to identify nearby genes and transcripts from the peaks obtained by MACS2. The HOMER software was used to discover de novo TF binding motifs within ATAC‐seq‐defined regions. An integrative genome viewer (IGV; http://igv.org/) was used to visualize the genome track corresponding to the gene locus.

### Chromatin Dynamics Analysis

Chromatin dynamics were analyzed by tracking telomere movement in three‐year normal healthy (GM00565) and HGPS fibroblast cell lines (AG06917) using the EGFP‐TRF2 plasmid. Briefly, 2.0 µg of EGFP‐TRF2 in Opti‐MEM (Thermo Fisher, 31985062) was mixed with 2.0 µL of Lipofectamine 3000 and 4.0 µL of P3000 transfection reagent in Opti‐MEM. The mixture was gently incubated for 20 min at RT, followed by a 4 h incubation at 37 °C and 5% CO_2_ in a humidified incubator. Next, transfected EGFP‐TRF2 cells were imaged at 0.07 s intervals for 5 min. Trajectory images were analyzed using the LIM Tracker Plugin in the Plugins menu of ImageJ, and MSD analysis was performed using a custom MATLAB script.

### Immunoblotting

Cellular lysates were prepared using RIPA buffer (Thermo Fisher Scientific, 89901) supplemented with a 1% protease inhibitor cocktail (Sigma, P8340). These preparations were then electrophoresed using SDS‐PAGE, and the resolved proteins were transferred to a polyvinylidene difluoride membrane (Thermo Fisher, LC2002). Nonspecific interactions were blocked using a protein‐based blocking reagent (Invitrogen, T2015) for 1 h. The membranes were then incubated with primary antibodies against H3K9me2/3 (CST, 5327), SUN1 (Proteintech, 24568), Nesprin 2 (Abcam, ab233034), and GAPDH (Sigma, G8795) overnight at 4 °C. Subsequently, membranes were incubated with the appropriate secondary antibodies, goat anti‐mouse IgG heavy and light chain antibody HRP Conjugated (Bethyl, A90‐116P), and goat anti‐rabbit IgG heavy and light chain antibody HRP Conjugated (Bethyl, A120‐101P) at RT for 1 h. Proteins were visualized using an ECL kit (Thermo Fisher Scientific, 32106).

### Proximity Ligation Assay (PLA) Analysis

Subconfluent cells were fixed on the glass bottom dish with 4% paraformaldehyde for 10 min at 4 °C and permeabilized with 0.1% Triton X‐100 for 10 min at RT. After blocking with Doulink Blocking Solution (Sigma‐Aldrich, DUO82007) for 60 min at 37 °C in a humidity chamber, samples were incubated overnight at 4 °C with anti‐SUN1 (1:200, Millipore, ABT273), anti‐Lamin A/C (1:200, Cell Signaling, 4777S), or anti‐Nesprin 2 (1:100, Sigma‐Aldrich, MABC86). Cells were washed with 1× wash Buffer A (Sigma‐Aldrich, DUO82049) and incubated in preheated humidity chamber for 1 h at 37 °C with anti‐PLUS and anti‐MINUS PLA probes diluted 1:5 in the Duolink Antibody Diluent (Sigma‐Aldrich, DUO92013). Samples were then incubated in a humidity chamber at 37 °C with ligation solution diluted 1:40 in the 1× ligation buffer. After 30 min, ligation buffer diluted 1:80 in 1× amplification buffer was added to the samples, followed by incubation for 100 min at 37 °C in preheated humidity chamber. Samples were mounted using Duolink in situ mounting medium (Sigma‐Aldrich, DUO82040) with DAPI. The cells were imaged using confocal laser microscopy through a 60× oil lens. To confirm the level of protein–protein interaction, the number and total intensity of red dots per cell were assessed based on the fluorescence signal.^[^ ^70^ ^]^

### siRNA‐Mediated Knockdown

To knockdown SUN1, siRNA targeting SUN1 (Santa Cruz Biotechnology, sc‐106672) and control siRNA (Santa Cruz Biotechnology, sc‐37007) were delivered to progerin‐expressing HeLa cells or HGPS fibroblast cells (Coriell Cell Repositories), according to the manufacturer's instructions. Briefly, 75 pmol siRNA in Opti‐MEM (Thermo Fisher, 31985062) and 7.5 µL Lipofectamine 3000 transfection reagent in Opti‐MEM were gently mixed and incubated for 5 min at RT. Progerin‐expressing HeLa cells at 70–80% confluency on glass bottom dishes were transiently transfected with siControl or siRNA targeting SUN1 for 24 h. After removing the transfection mixture and replacing it with fresh growth medium, cells were immunostained for analysis.

### Data Processing and Statistical Analysis

All statistical analyses were performed using GraphPad Prism (GraphPad software, USA), and statistical significance was analyzed using unpaired *t*‐test or Student's *t*‐test when two groups were compared. One‐way analysis of variance (ANOVA) using Tukey's test or Bonferroni's post hoc test was used for pairwise comparisons between multiple groups. Error bars represent the standard deviation (S.D.) or standard error of the mean (S.E.M.), as indicated. Significance levels are indicated with asterisks in each figure as follows: *: *p* < 0.01; **: *p* < 0.005; ***: *p* < 0.001; ****: *p* < 0.0001.

## Conflict of Interest

The authors declare no conflict of interest.

## Author Contributions

J.P., J.J., and K.X. contributed equally to this work. J.P. and J.J. performed and analyzed all experiments unless otherwise specified and cowrote the manuscript. K.X. developed computational model to simulate the nuclear wrinkling. S.L. and S.H. analyzed the protein contents and chromatin dynamics in the human dermal fibroblasts. J.J., Y.L., and W.C. developed the Tet‐On system. W.C., B.L., and S.H.K. provided a conceptual design of the experimental settings. K.X. and B.L. cowrote the manuscript. D.K. supervised the project, designed the experiments, and wrote the manuscript.

## Supporting information



Supporting Information

Supplemental Movie 1

Supplemental Movie 2

Supplemental Movie 3

Supplemental Movie 4

## Data Availability

All data needed to evaluate the conclusions in the paper are present in the paper and/or the Supporting Information.
