# Supplementary material for: Attenuated Nuclear Tension Regulates Progerin‐Induced Mechanosensitive Nuclear Wrinkling and Chromatin Remodeling
Source: Adv Sci (Weinh). 2025 May 8;12(31):2502375. doi: 10.1002/advs.202502375 (PMC12376529; doi:10.1002/advs.202502375)

# Attenuclear tension regulates progerin-induced mechanosensitive nuclear wrinkling and chromatin remodeling

#

**Ji-Eun Park^1,#^, Juhyeon Jo^1,#^, Kun Xu^2,#^, Sun-Ah Lee^1^, Seong-Beom Han^1^, YigJi Lee^3^, Won-Ki Cho^3^, Bo Li^2^, Soo Hyun Kim^1,4^, and Dong-Hwee Kim^1,4,5,*^**

^1^ KU-KIST Graduate School of Converging Science and Technology, Korea University, Seoul, 02841, S. Korea

^2^ Department of Engineering Mechanics, Tsinghua University, Beijing 100084, China

^3^ Department of Biological Sciences, Korea Advanced Institute of Science and Technology (KAIST), Daejeon, 34141, S. Korea.

^4^ Biomaterials Research Center, Biomedical Research Division, Korea Institute of Science and Technology (KIST), Seoul 02792, S. Korea

^5^ Department of Integrative Energy Engineering, College of Engineering, Korea University, Seoul, 02841, S. Korea

**^#^** Equally contributed to this work

***** The supplementary information includes following data *****

**Supplementary Figure: 1, 2, 3, 4, 5, 6, 7, 8, 9, 10**

**Supplementary Figure legends**

**Supplementary Movie: 1, 2, 3, 4 (still images)**

**Supplementary Movie lege****nds**

Fig. S1.


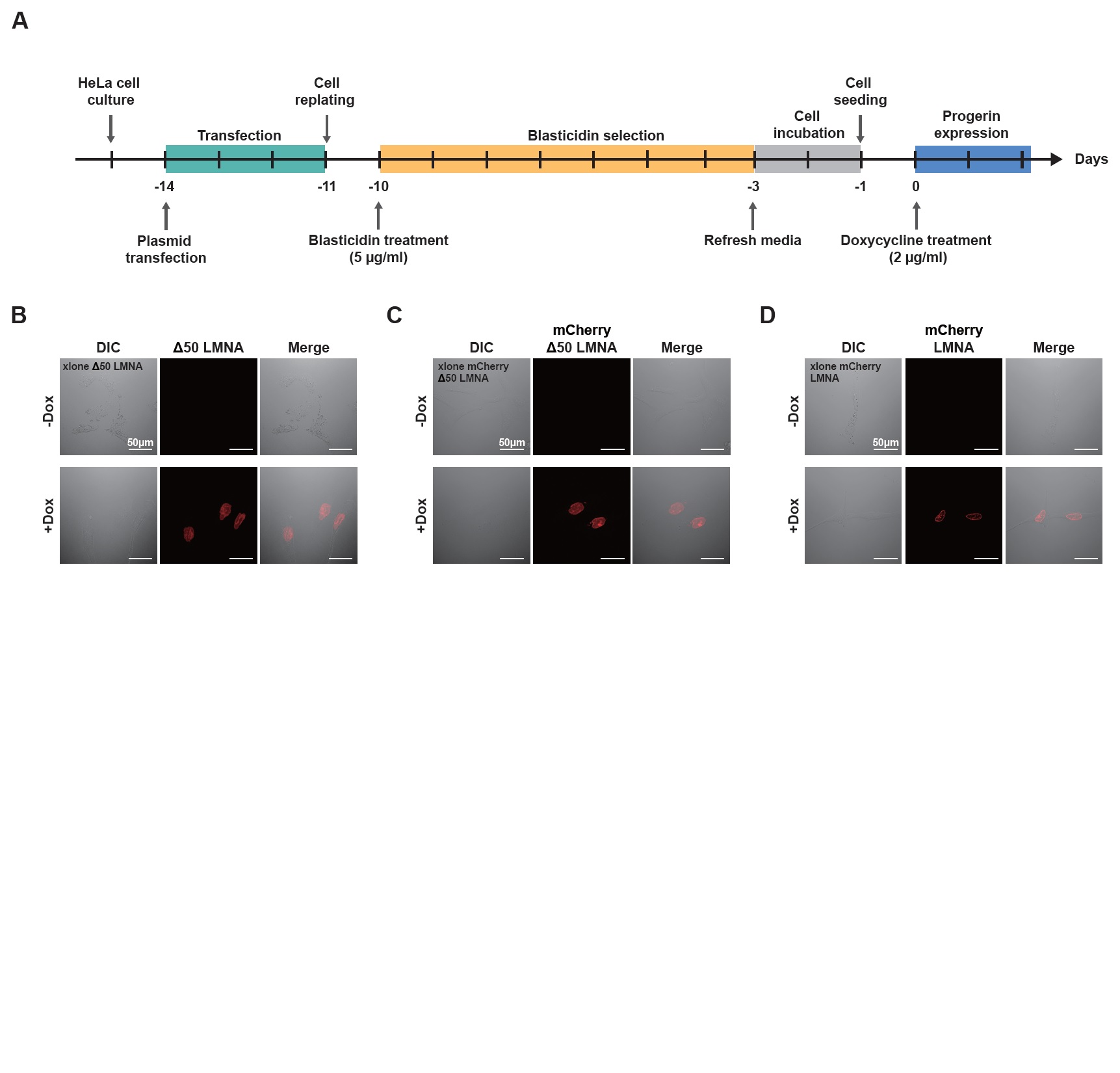


**Fig. S1. Preparation of doxycycline-induced progerin-expressing HeLa cells.** **A.** Schematic illustration of experimental time line used to generate HeLa cells with the Tet-On system for doxycycline-inducible gene expressing. Refer to the Methods section for details. **B–D.** Confirmation of doxycycline-controlled progerin or lamin A/C expression in HeLa cells. Tet-On HeLa cells transfected with Δ50 LMNA (B), mCherry-tagged Δ50 LMNA (C), and mCherry-tagged control LMNA (D) were imaged before (**–**Dox) and after (+Dox) doxycycline treatment. To confirm the doxycycline-induced progerin expression, Δ50 LMNA-transfected cells were immunostained with the progerin antibody (red, B).

Fig. S2.


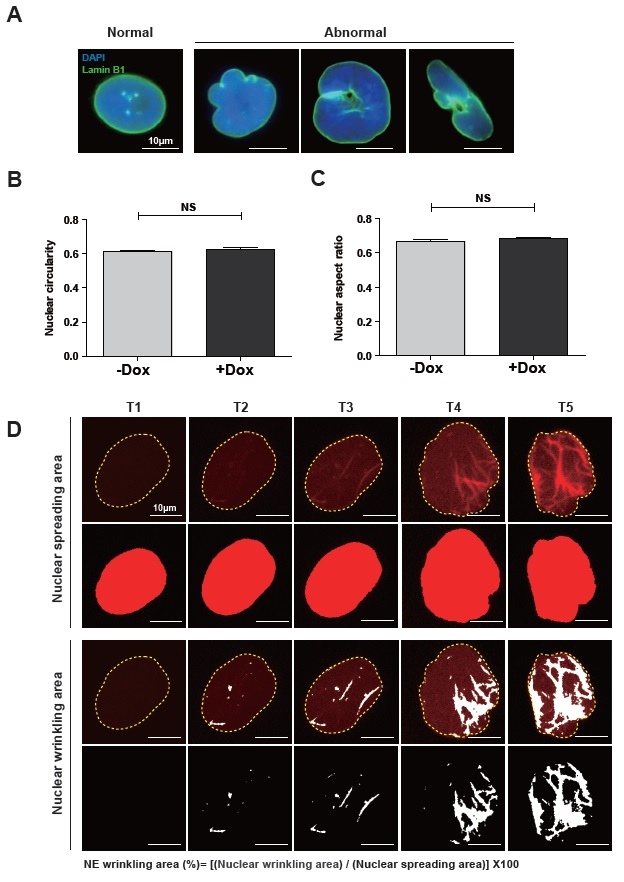


**Fig. S2. Defining progerin-induced nuclear deformation. A–C.** Quantification of abnormal nuclear morphology in progerin-expressing HeLa cells. Nuclei immunostained for lamin B1 (green) and counterstained with DAPI (blue) depict the normal and abnormal nuclear morphology in doxycycline-untreated control and doxycycline-induced progerin-expressing Tet-On HeLa cells, respectively (A). Note that doxycycline-induced progerin-expressing cells feature abnormal nuclear shape featuring blebs, lobulation, and surface wrinkling compared to control cells displaying a normal round nuclear shape. Doxycycline-induced progerin expression had no significant effect traditional nuclear morphometric parameters such as circularity (B) and aspect ratio (C), defined as 4π (area)/(perimeter^2^) and the ratio of the longest axis to its perpendicular shortest axis, respectively. In panels B**–**C, >130 nuclei were analyzed for each condition; error bars indicate the S.E.M.; and unpaired t-test was applied for comparing two groups (NS: not significant). **D.** Defining nuclear wrinkling. Magnitude of nuclear envelope (NE) wrinkling was estimated by the nuclear surface area occupied by NE wrinkling area, where the nuclear surface area and NE wrinkling area indicate a nuclear spreading area (yellow dotted lines filled with red color) and a faction of nuclear surface representing the oversaturated fluorescence intensity (marked with white), respectively. Note that doxycycline-controlled progerin expression induced a time-dependent progression of NE wrinkling.

Fig. S3.


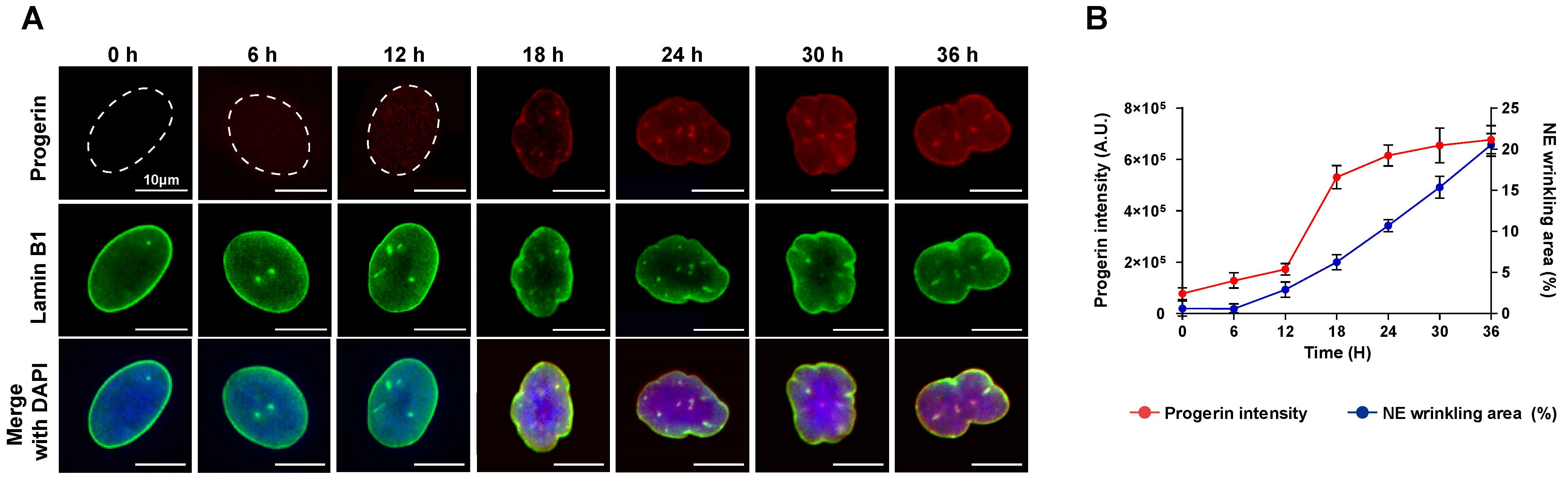


**Fig. S3. Time-dependent evolution of NE wrinkling in doxycycline-induced progerin-expressing cells. A.** Representative immunostained nuclei of doxycycline-inducible progerin-expressing Tet-On HeLa cells. Progerin-expressing cells were immunostained for progerin (red), lamin B1 (green), and nucleus (DAPI, blue) after doxycycline treatment from 0 h to 36 h. **B.** Measurement of progerin expression and NE wrinkling. Doxycycline-induced progerin-expressing cells were immunostained every 6 h for 36 h, where abrupt increase of progerin expression was followed by NE wrinkling. In panel B, > 20 nuclei were analyzed at each time point; error bars indicate the S.E.M.

Fig. S4.


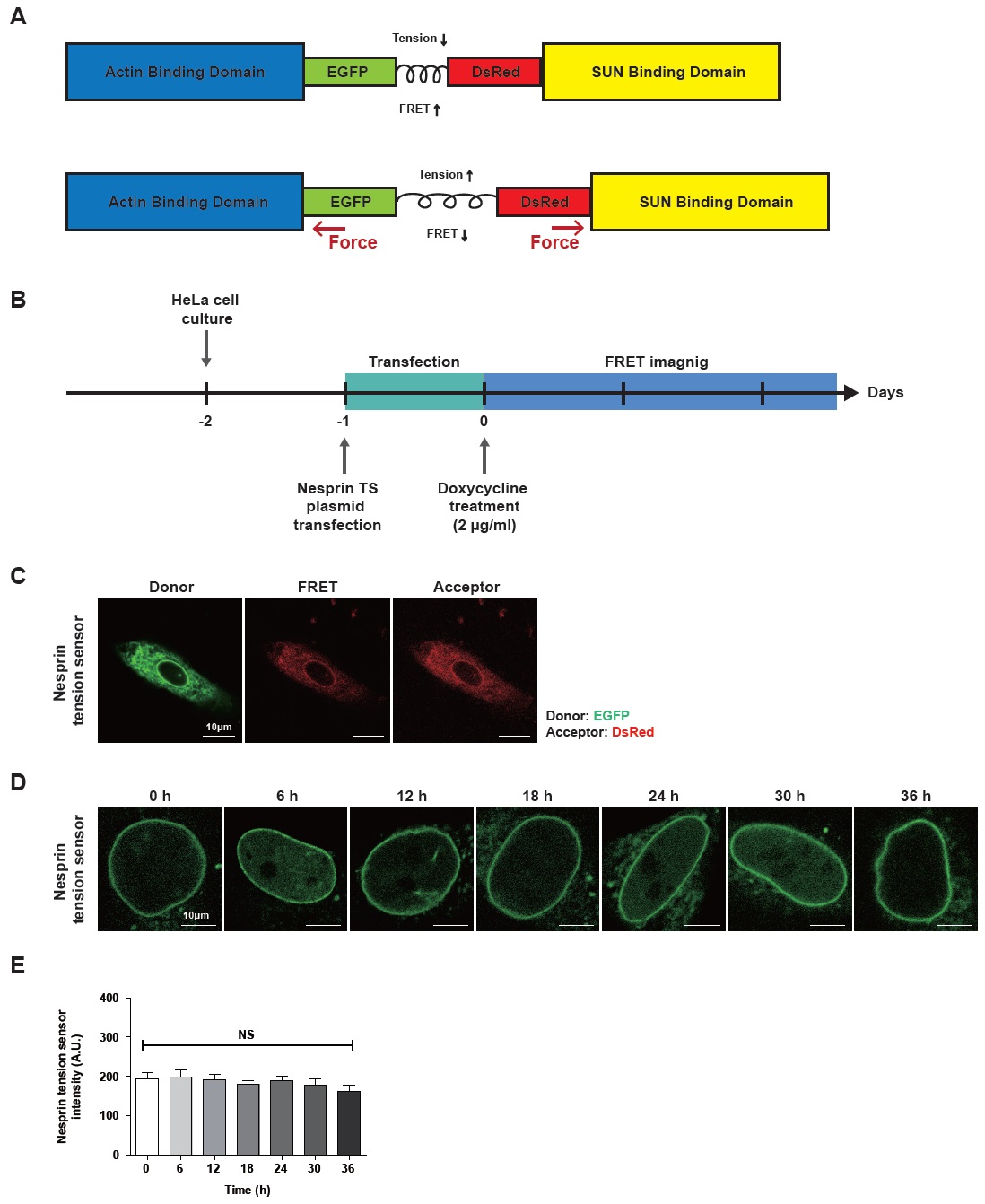


**Fig. S4. Nesprin tension sensor-based monitoring of NE tension.** **A.** Schematic illustration of nesprin tension sensor. Fluorescence energy transfer (FRET)-based NE tension sensor module tagging the inner nuclear membrane SUN-binding domain and cytoplasmic F-actin-binding domain mimics the LINC complex associating nesprin 2. **B.** Experimental outline of transfection of Tet-On HeLa cells with nesprin tension sensors. Refer to the Methods section for details. **C.** Representative fluorescent confocal images of nesprin tension sensor-transfected progerin-expressing Tet-On HeLa cells. FRET signal was detected through the emission of DsRed (acceptor) in response to excitation of EGFP (donor). **D-E.** Time-dependent monitoring of fluorescent intensity of nesprin tension sensor after doxycycline-induced progerin expression. Representative fluorescence images display the expression of EGFP-tagged donor side of nesprin tension sensors along the nuclear envelope (D). No significant decay in the fluorescent intensity of the nesprin tension sensors was observed after doxycycline treatment (E). In panel E, > 20 cells were analyzed per condition; error bars indicate the S.E.M.; and one-way ANOVA using Bonferroni’s post-hoc test was applied to compare all possible pairs of conditions (NS: not significant).

Fig. S5


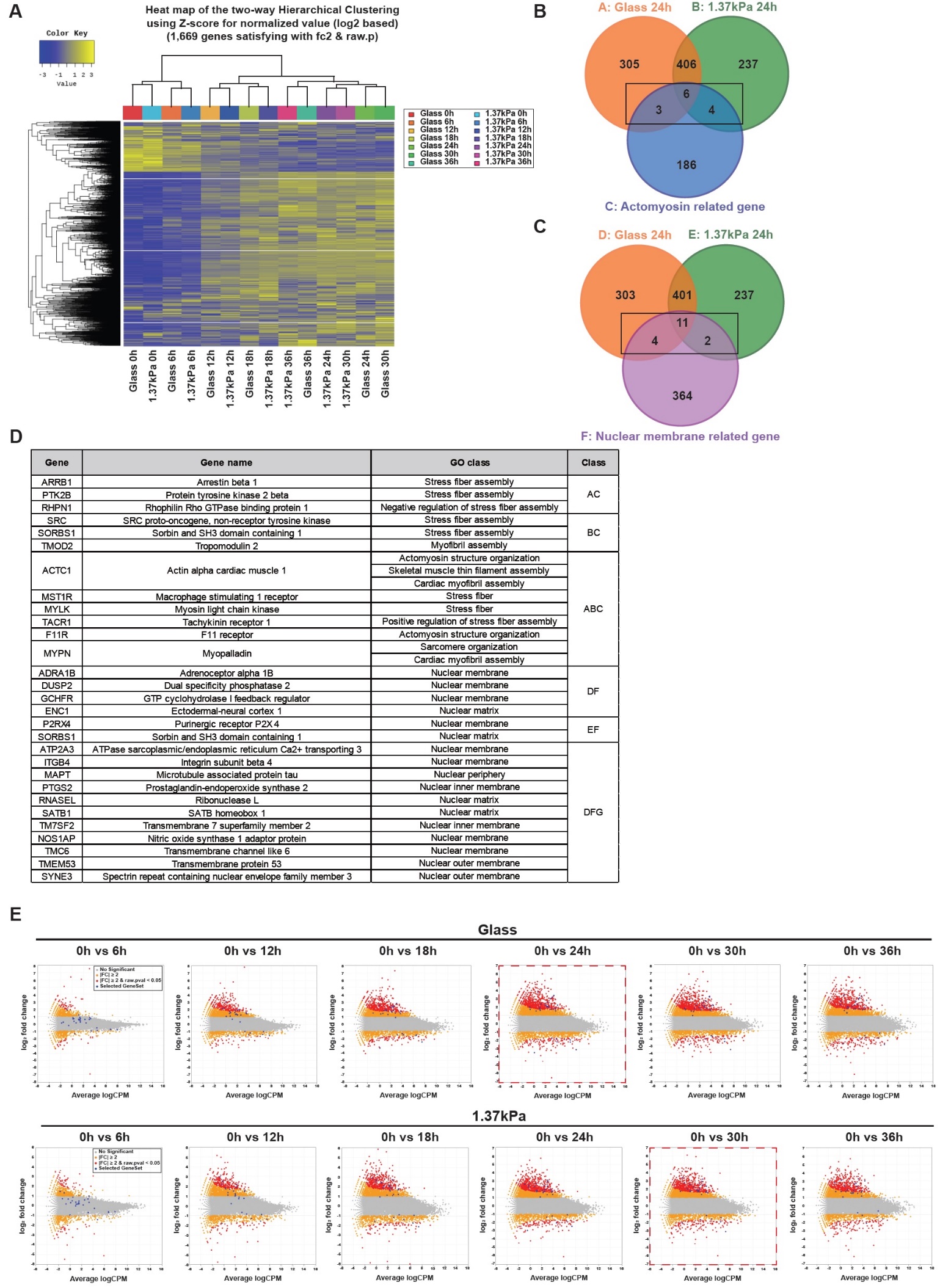


**Fig. S5. Substrate stiffness-dependent differences in the expression of actomyosin and nuclear membrane associated genes. A.** Differential gene expression in response to progerin expression depending on substrate stiffness. RNA sequencing data was plotted by heat map of the two-way hierarchical clustering using Z-score for normalized value. Hierarchical clustering analysis (Euclidean method, complete linkage) was performed on significant data to group samples and genes with similar expression levels (normalized values), where values ​​are corrected by FPKM (Fragments Per Kilobase of transcript per Million mapped reads) and TPM (Transcripts Per Kilobase Million). **B-E.** The Venn diagrams summarize the number of DEGs in each category, comparing data from doxycycline-treated 24 h on glass and 1.37 kPa substrates with actomyosin-related genes (B) and nuclear membrane-related genes (C). Each dataset was analyzed based on the criteria of fold change ≥ |2.0| and raw p-value < 0.05. Gene list corresponding to the intersection in the Venn diagram of actomyosin-related genes and nuclear membrane-related genes (D). MA plot comparing the untreated groups (denoted by Glass 0 h and 1.37 kPa 0 h) and the groups treated with doxycycline for 36 h at 6 h intervals, focusing on actomyosin and nuclear membrane-related genes (E). In panel E, colored red is the standard for fold change ≥ |2.0| and the raw p-value < 0.05, colored yellow is the standard for fold change ≤ |2.0|, colored blue is the actomyosin and nuclear membrane related genes, and colored gray is no significant.

Fig. S6


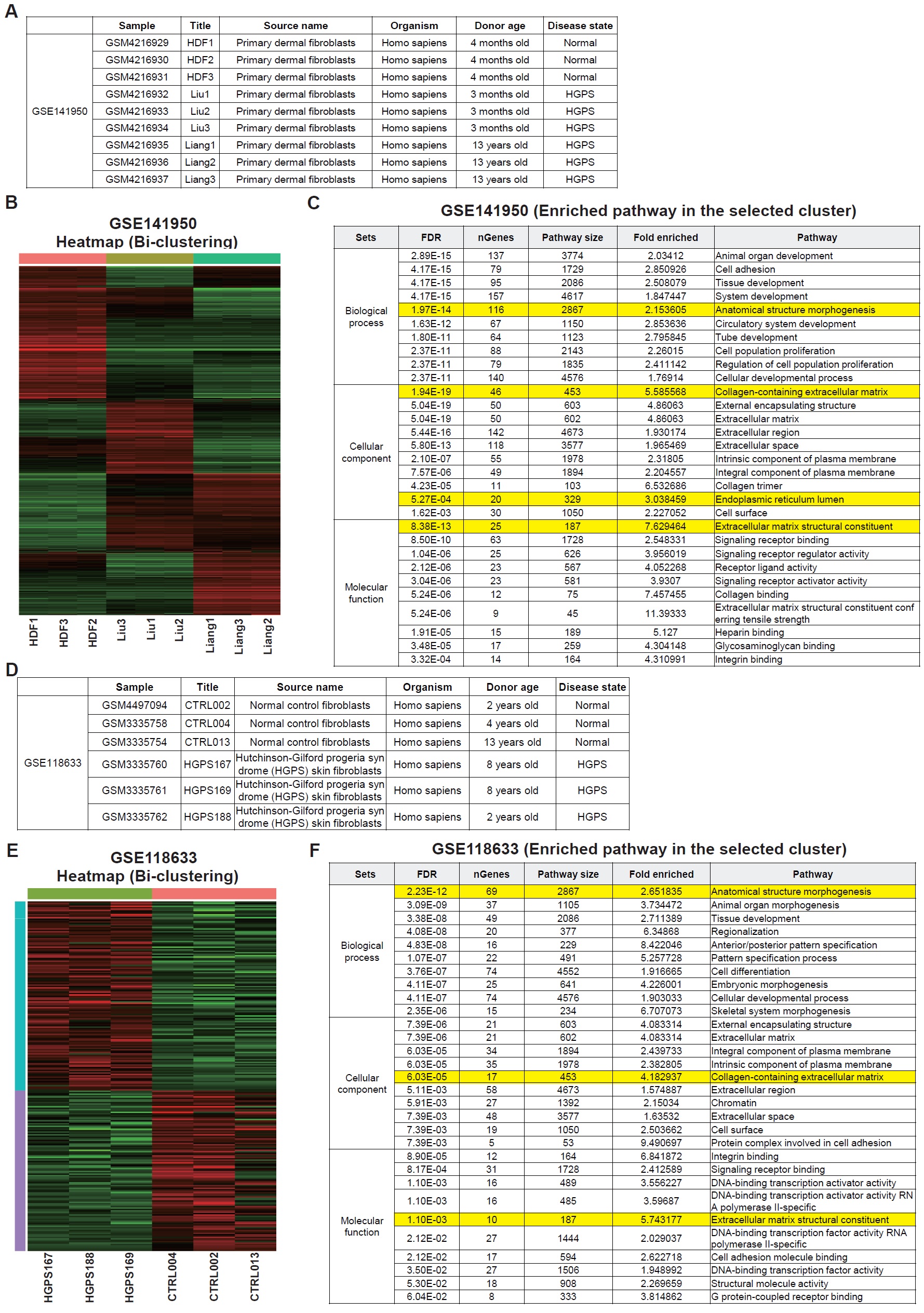


**Fig. S6. RNA Sequencing of primary dermal fibroblasts from HGPS Patients. A-F.** Gene expression analysis using RNA sequencing results from GEO (Gene Expression Omnibus) comparing healthy individuals and HGPS patients. Sample information analyzed using GSE141950 (Title: RNA-seq analysis of primary dermal fibroblasts in Hutchinson-Gilford progeria syndrome) (A), bi-clustering heatmap results among groups (B), and enriched pathway analysis results (C). The yellow highlights indicate results that correspond to those shown in Figure 4D. In panels B and C, data were analyzed using iDEP (Integrated Differential Expression and Pathway analysis). Sample information analyzed using GSE118633 (Title: SAMMY-seq, H3K9me3 and H3K27me3 ChIP-seq and RNA-seq of control and progeria fibroblasts) (D), bi-clustering heatmap results among groups (E), and enriched pathway analysis results (F). The yellow highlights indicate results that correspond to those shown in Figure 4D. In panels E and F, data were analyzed using iDEP (Integrated Differential Expression and Pathway analysis).

Fig. S7


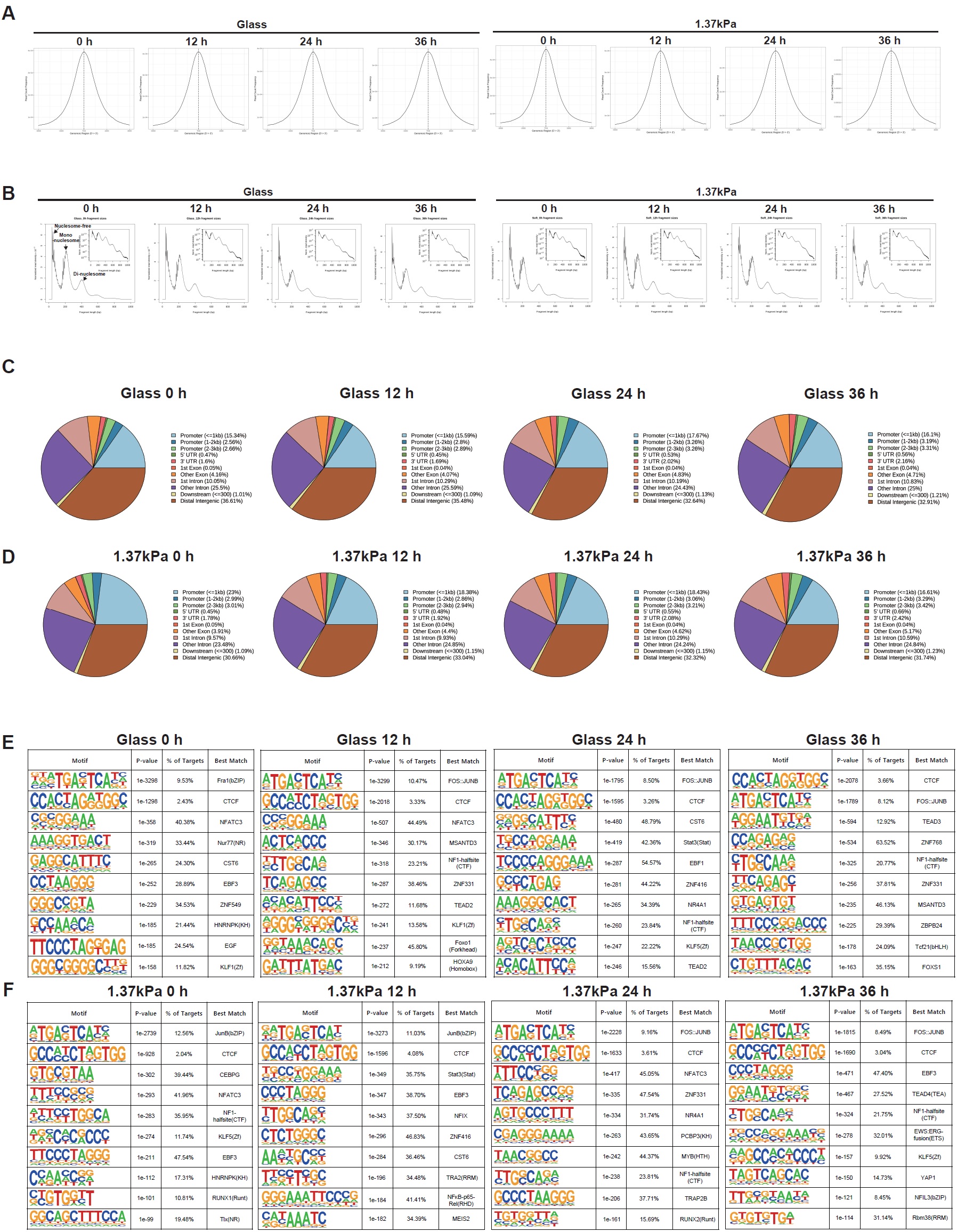


**Fig. S7.** **ATAC sequencing of progerin expression across different substrate stiffness levels. A-D.** Quality assessment of ATAC sequencing data. Read counts are enriched at the transcription start site (TSS) within genomic regions encompassing ± 3 kb from the TSS in samples treated with doxycycline for 36 hours at 12-hour intervals on glass substrate (A) and 1.37 kPa substrate (B). Proportions of peaks associated with specific functional categories (e.g., introns, exons, promoters, etc.) are visualized in the pie charts for glass substrate (C) and 1.37 kPa substrate (D). **E-F.** The top 10 de novo TF binding motifs identified among progerin-expressing cell populations were validated in glass substrate (E) and 1.37 kPa substrate (F) treated with doxycycline for 12 h, using Homer software.

Fig. S8


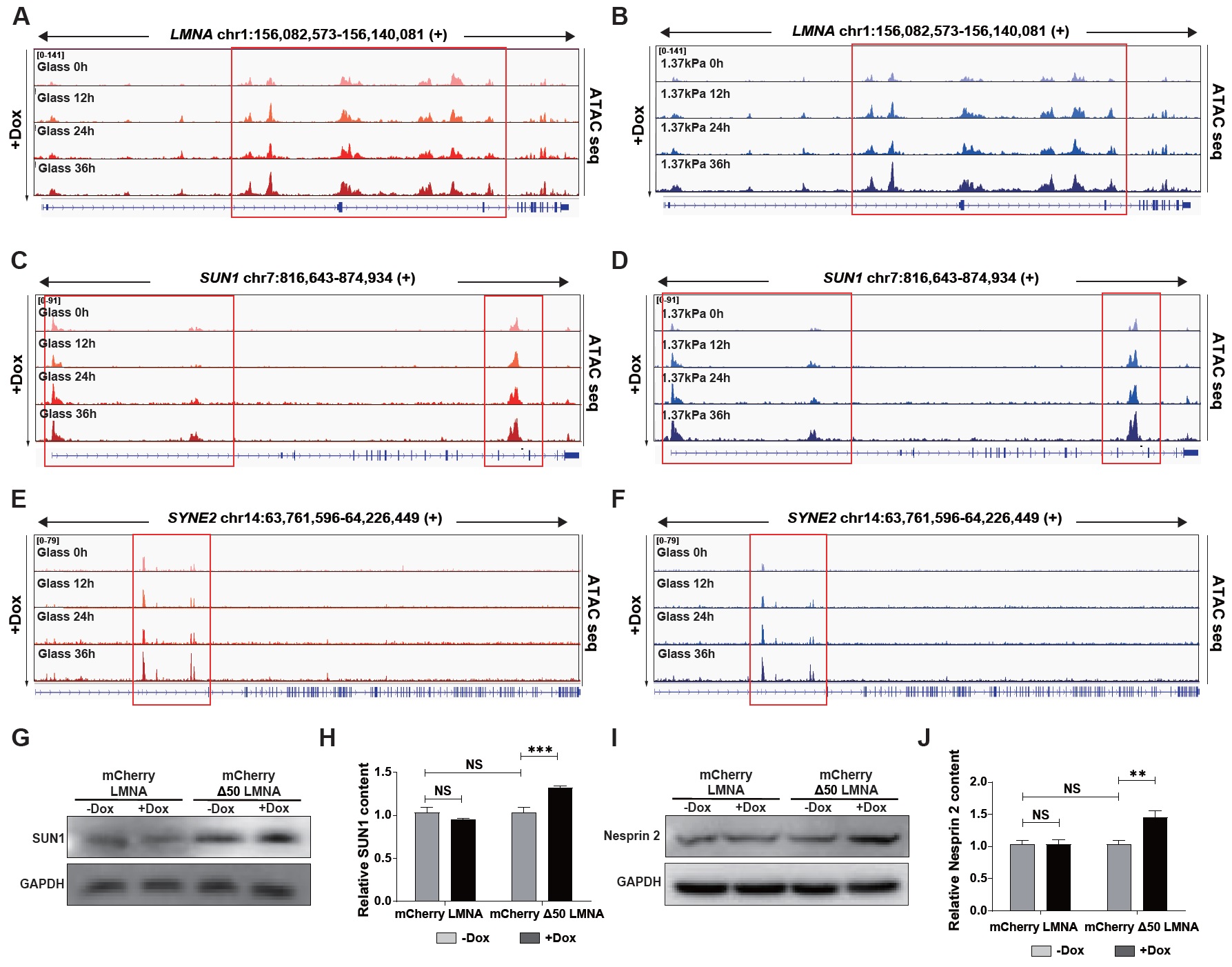


**Fig. S8. Alterations in LMNA, SUN1, and SYNE2 gene expression in progerin-expressing cells by ATAC sequencing and RNA sequencing. A-F.** Visualization of ATAC sequencing and RNA sequencing peaks for progerin-expressing cells treated with doxycycline for up to 36 hours on control glass and soft PAG substrates. Red and blue peaks indicate stiff glass and soft PAG substrates, respectively. Red box regions in the full genes of LMNA (A, B), SUN1 (C, D), and SYNE2 (E, F) were depicted in Fig. 7A-F. **G-J.** Confirmation of SUN1 and nesprin 2 expression. Protein content was detected and quantified by immunoblotting with antibodies to SUN1, nesprin 2, and GAPDH in doxycycline-induced expression of mCherry-tagged LMNA and mCherry-tagged Δ50 LMNA. Doxycycline-induced expression of mCherry-tagged LMNA did not alter total SUN1 protein levels, whereas doxycycline-induced expression of mCherry-tagged Δ50 LMNA significantly increased total SUN1 protein levels (G, H). Similarly, mCherry-tagged LMNA did not change the expression of nesprin 2, while mCherry-tagged Δ50 LMNA significantly increased nesprin 2 expressions (I, J). In panels H and J, three independent experiments were performed; error bars indicate the S.E.M.; and one-way ANOVA using Tukey's test was applied for comparison between groups (***: *p* < 0.001, **: *p* < 0.05, NS: not significant).

Fig. S9


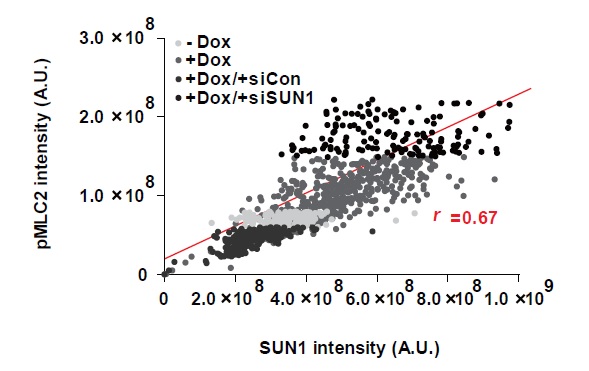


Fig. S9. Correlative analysis of SUN1 and pMLC2 expression. The Pearson product-moment correlation assessment showed a highly correlative relationship between SUN1 and pMLC2 expression levels (*r* = 0.67), where correlation assessment and linear regression were applied to the merged dataset regardless of each condition. Datasets were adapted from Fig. 5B and C, where > 150 cells were analyzed per condition

Fig. S10


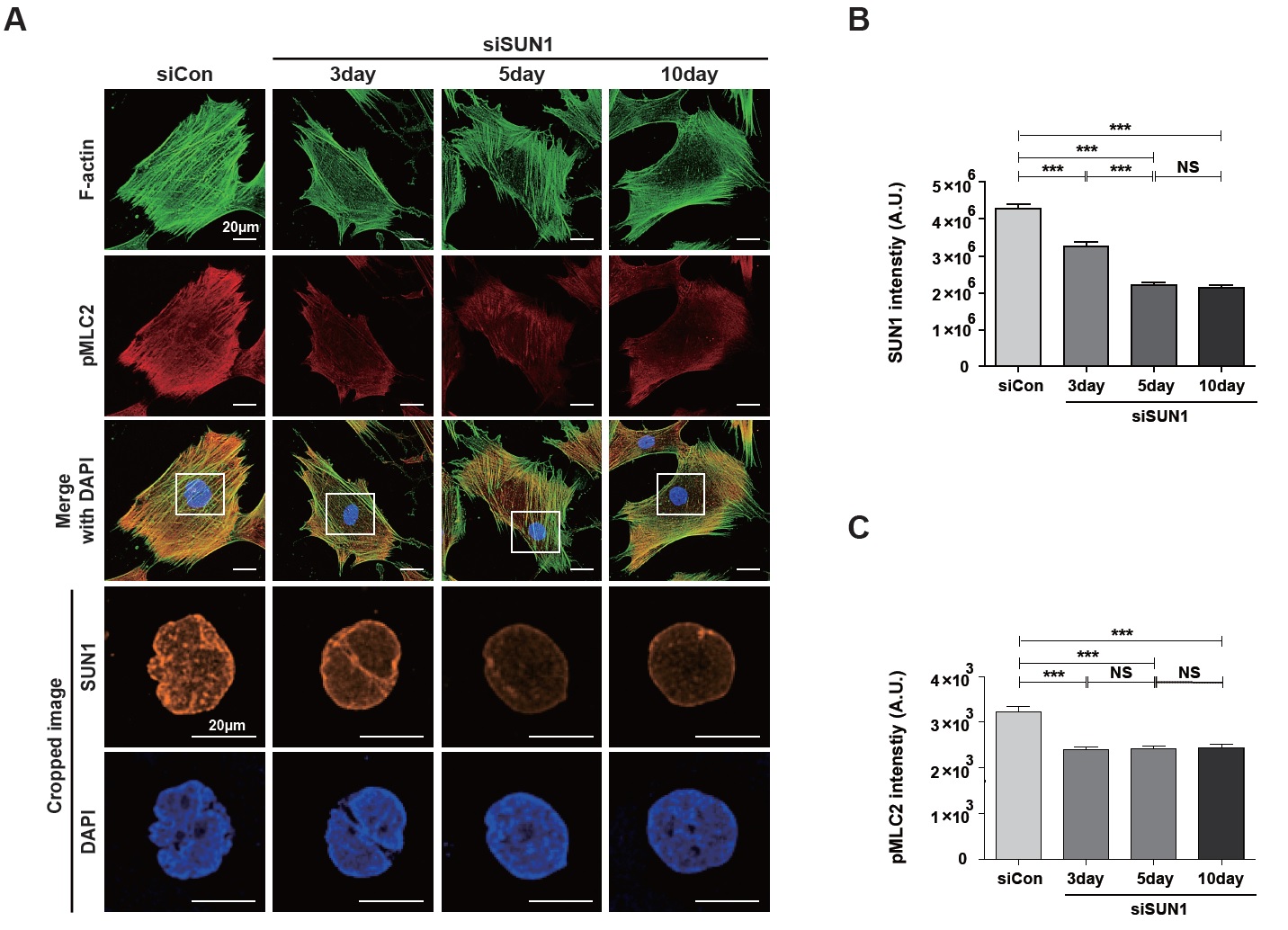


**Fig. S10. Temporal changes of SUN1 and pMLC2 expression in response to SUN1 depletion. A.** Representative immunofluorescence images showing F-actin (green), pMLC2 (red), SUN1 (orange), and nucleus (DAPI, blue) in siControl-transfected HGPS fibroblasts or HGPS cells transfected with siSUN1 followed by 3, 5, and 10 days of incubation. Note that F-actin organization largely remained intact. **B–C.** Quantification of SUN1 and pMLC2 expression. Compared to siControl-transfected HGPS fibroblasts, a significant reduction in SUN1 and pMLC2 expression was observed in siSUN1-transfected cells after 3 days and maintained after 5 days. In panels B and C, > 20 cells were analyzed per condition; error bars indicate the S.E.M.; and one-way ANOVA using Tukey's test was applied for comparison between groups (***: *p* < 0.001, NS: not significant).

**Movie S1. Time-lapse monitoring of progerin expression and NE wrinkling in mCherry-tagged progerin-expressing Tet-On HeLa cells placed on control glass, stiff PAG, and soft PAG substrates.** Live cell imaging was performed every 20 min for 36 h after doxycycline treatment. Red represents the mCherry signal. Progerin expression and NE wrinkling in response to doxycycline treatment were delayed on soft PAG (E ~1.37 kPa) substrates (right) compared to glass (left) and stiff (E ~34 kPa) PAG substrates (middle). Movies correspond to images shown in Fig. 1G**–**I.

**
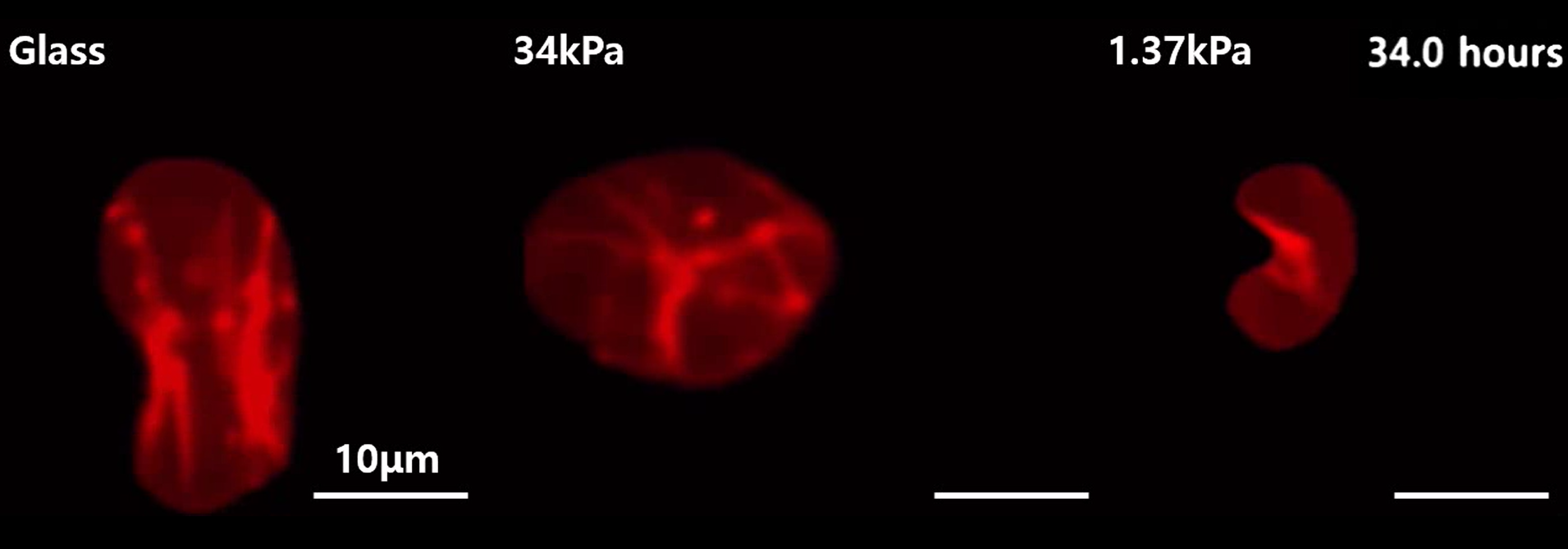
**

**Movie S2. Time-lapse monitoring of 3-D progression of NE wrinkling in mCherry-tagged progerin-expressing Tet-On HeLa cells placed on control glass, stiff PAG, and soft PAG substrates.** Live imaging was performed every 30 min for 16 h followed by an 18 h incubation with doxycycline. NE wrinkling by doxycycline-induced progerin expression was delayed in cells placed on soft PAG substrates (right) compared to those placed on glass (left) and stiff PAG substrates (middle). To depict the evolution of NE wrinkling 3D reconstructed depth-coding images were presented in a cross-section of the nuclei.

**
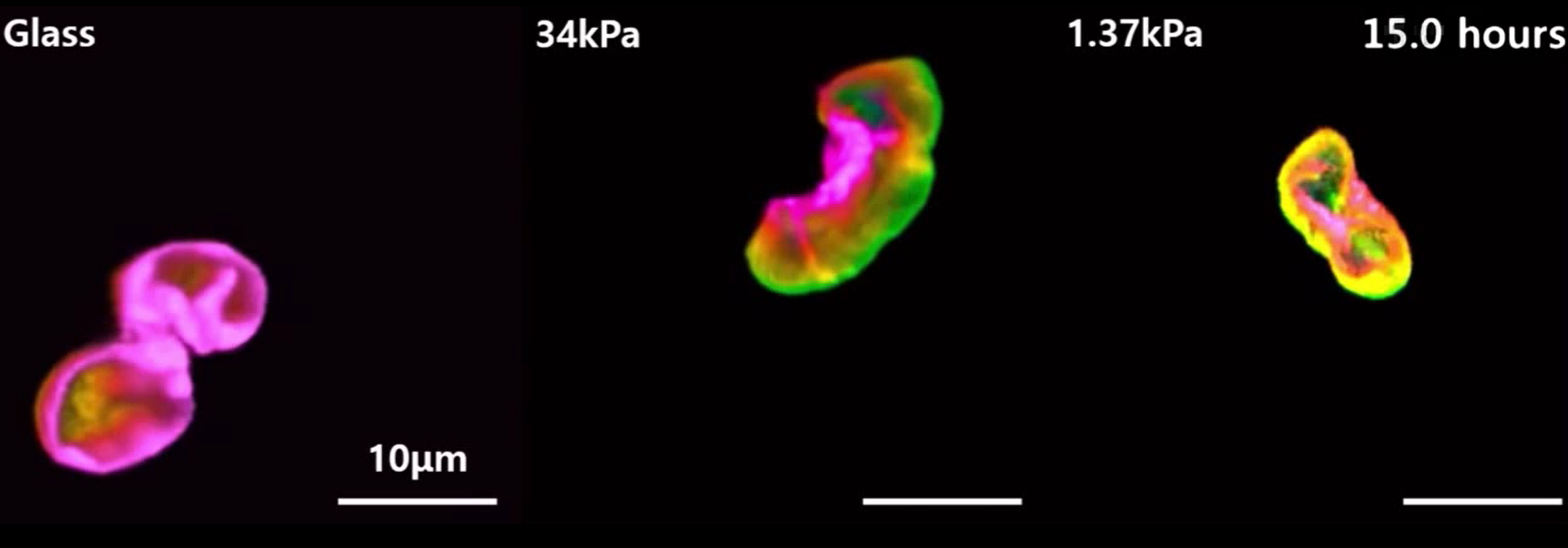
**

**Movie S3. Simulation of nuclear wrinkling in response to different substrate stiffness.** Nuclear wrinkling occurs in a time-dependent manner following nuclear volume changes, with the fastest onset observed under stiff conditions (left), followed by medium (middle), and soft (right) conditions. Movies correspond to images shown in Fig. 3B.


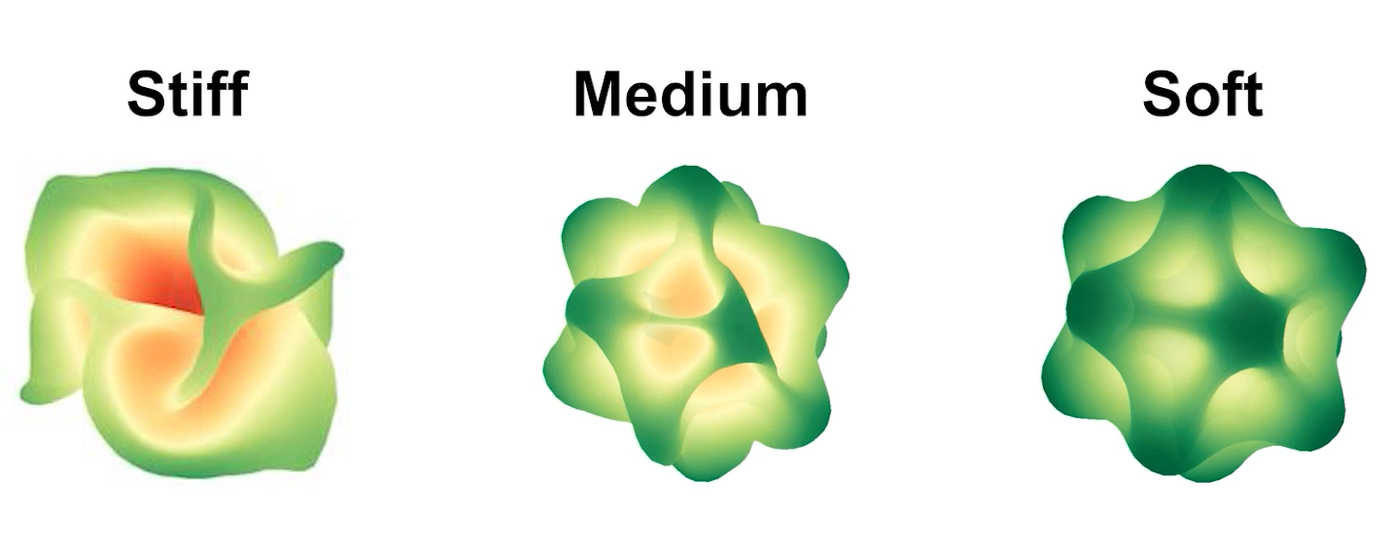


**Movie S4. Time-lapse monitoring of chromatin dynamics.** Chromatin dynamics in healthy control group and HGPS patients were assessed by tracking TRF2-GFP particles. Live imaging was conducted for 5 min at 0.07 sec intervals, revealing an increased movement of TRF2-GFP particles in 3YR (HGPS) compared to 3YR (Control). Movies correspond to images shown in Fig. 6A-C.


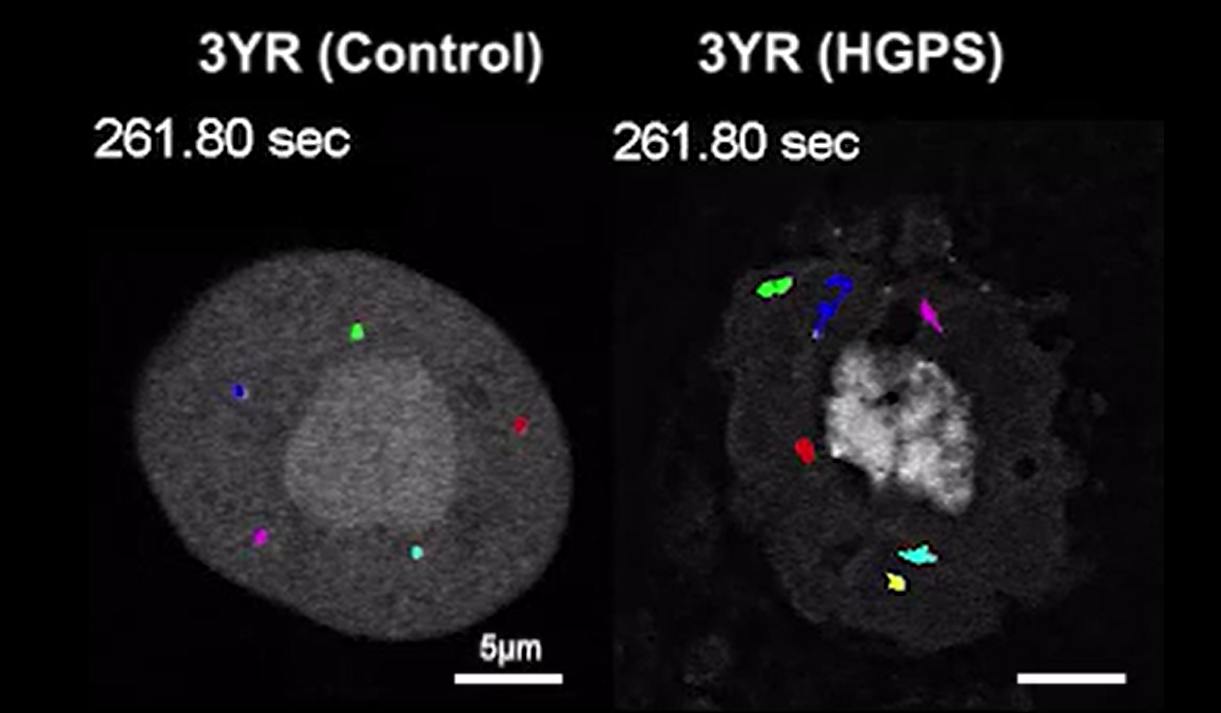

Supplement: Supplementary file 1 — Supporting Information [file ADVS-12-2502375-s002.docx]
